# Exploring the feasibility of using long-term stored newborn dried blood spots to identify metabolic features for congenital heart disease screening

**DOI:** 10.1186/s40364-023-00536-y

**Published:** 2023-11-13

**Authors:** Scott R. Ceresnak, Yaqi Zhang, Xuefeng B. Ling, Kuo Jung Su, Qiming Tang, Bo Jin, James Schilling, C. James Chou, Zhi Han, Brendan J. Floyd, John C. Whitin, Kuo Yuan Hwa, Karl G Sylvester, Henry Chubb, Ruben Y. Luo, Lu Tian, Harvey J. Cohen, Doff B. McElhinney

**Affiliations:** 1 Department of Pediatrics, Stanford University School of Medicine, Stanford, CA 94305 USA; 2 College of Automation, Guangdong Polytechnic Normal University, 293 Zhongshan Avenue West, Tianhe District, Guangzhou, 510665 China; 3 Department of Surgery, Stanford University School of Medicine, Stanford, CA 94305 USA; 4 mProbe Inc, Palo Alto, CA 94303 USA; 5 The Center for Biomedical Industries, National Taipei University of Technology, Taipei, Taiwan; 6 Department of Pathology, Stanford University School of Medicine, Stanford, CA 94305 USA; 7 Department of Biomedical Data Science, Stanford University School of Medicine, Stanford, CA 94305 USA; 8 Departments of Cardiothoracic Surgery, Stanford University School of Medicine, Stanford, CA 94305 USA

**Keywords:** Congenital Heart Disease, Screening and classification, Metabolite profiling, Dried blood spot, LC-MS/MS

## Abstract

Congenital heart disease (CHD) represents a significant contributor to both morbidity and mortality in neonates and children. There’s currently no analogous dried blood spot (DBS) screening for CHD immediately after birth. This study was set to assess the feasibility of using DBS to identify reliable metabolite biomarkers with clinical relevance, with the aim to screen and classify CHD utilizing the DBS. We assembled a cohort of DBS datasets from the California Department of Public Health (CDPH) Biobank, encompassing both normal controls and three pre-defined CHD categories. A DBS-based quantitative metabolomics method was developed using liquid chromatography with tandem mass spectrometry (LC-MS/MS). We conducted a correlation analysis comparing the absolute quantitated metabolite concentration in DBS against the CDPH NBS records to verify the reliability of metabolic profiling. For hydrophilic and hydrophobic metabolites, we executed significant pathway and metabolite analyses respectively. Logistic and LightGBM models were established to aid in CHD discrimination and classification. Consistent and reliable quantification of metabolites were demonstrated in DBS samples stored for up to 15 years. We discerned dysregulated metabolic pathways in CHD patients, including deviations in lipid and energy metabolism, as well as oxidative stress pathways. Furthermore, we identified three metabolites and twelve metabolites as potential biomarkers for CHD assessment and subtypes classifying. This study is the first to confirm the feasibility of validating metabolite profiling results using long-term stored DBS samples. Our findings highlight the potential clinical applications of our DBS-based methods for CHD screening and subtype classification.

**Supplementary Information:**

The online version contains supplementary material available at 10.1186/s40364-023-00536-y.

To the editor,

Prenatal diagnosis and early detection advancements have contributed to a gradual decline in the mortality rate associated with congenital heart disease (CHD) in children [1, 2]. However, the methodologies for the detection of cyanotic CHD exhibit less than 75% sensitivity in detecting critical CHD [3, 4]. Currently, there is no comprehensive, cost-effective screening method available at birth that can reliably and consistently detect the diverse range of CHD conditions. Meanwhile, millions of infants in the United States undergo newborn screening (NBS), where substances in dried blood spots (DBS) are measured to check for certain genetic, endocrine, and metabolic disorders [5]. Despite this, no DBS newborn screening exists for CHD at birth.

Our core hypothesis proposes that comprehensive metabolic profiling of a long-term stored DBS at birth through liquid chromatography-mass spectrometry (LC-MS) could model and assess cardiac and other organ anomalies with high precision [6]. We developed an LC-MS based metabolic screening method (Figure S1), to construct a baseline for neonate DBS metabolites and identify a biomarker panel as a molecular surrogate to assess congenital cardiac abnormalities.

To assess the feasibility of using long-term stored DBS for CHD biomarker identification, we constructed a cohort of 20 neonates (5 controls and 15 CHD patients). The 15 CHD patients comprised 4 diagnosed with CHD-TOF (Tetralogy of Fallot), 5 with CHD-IAS (2 Brugada, 3 Long QT syndrome), and 6 with CHD-CMP (3 dilated, 3 hypertrophic cardiomyopathy) (Table S1). We reassessed the concentrations of 28 NBS metabolites commonly found in California Department of Public Health (CDPH) NBS records in these DBS samples stored at -20 °C for up to 15 years (Figure S2). 24 out of the 28 metabolites exhibit a strong correlation, affirming both the robustness of our metabolomic profiling workflow and the reliability of these DBS samples after many years of storage (Fig. 1).

**Fig. 1:**
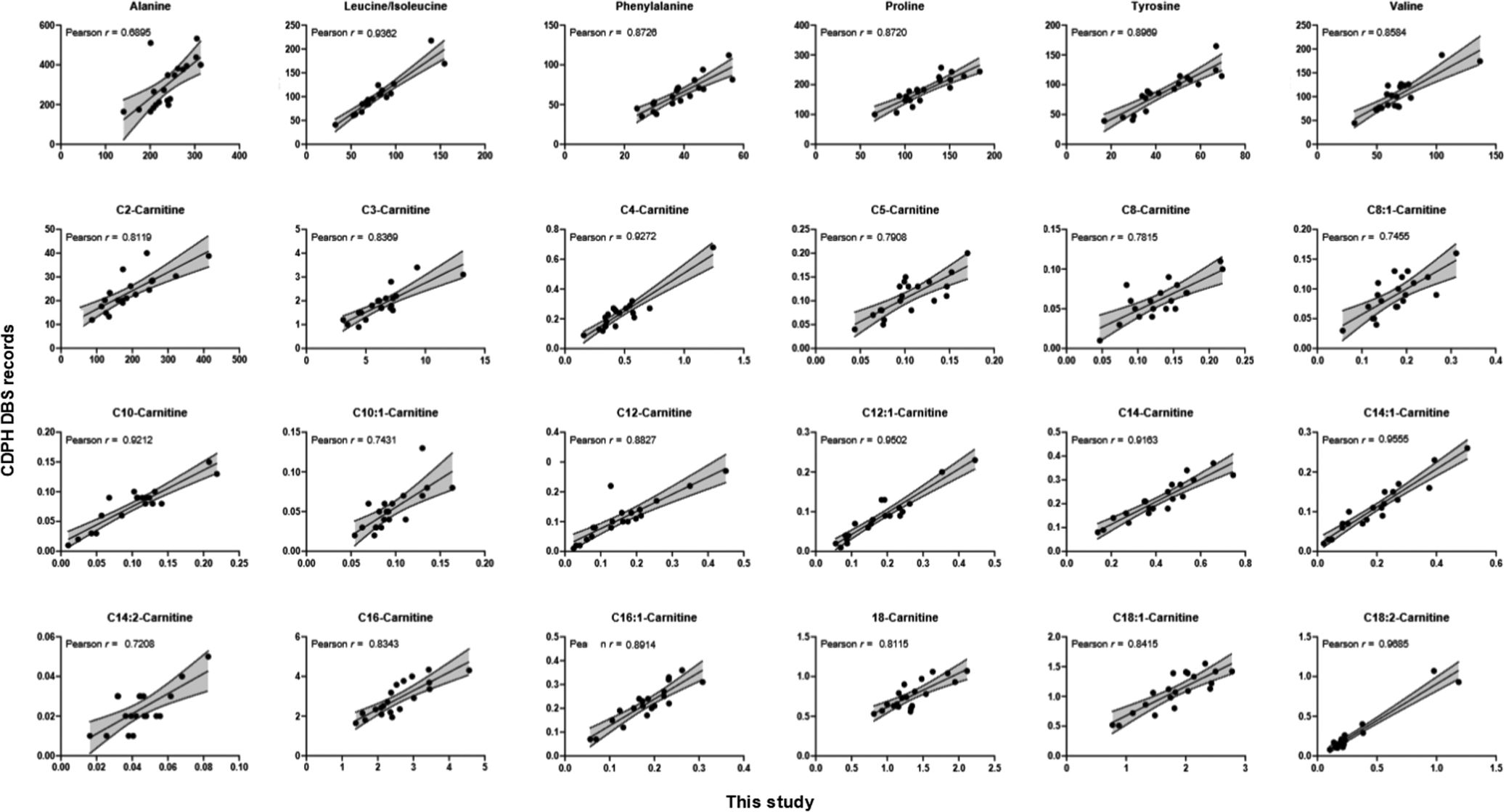
Impact of the time storage of metabolites. Scatter plots showing the positive correlations of metabolic profiling between this study (X-axis) and the CDPH DBS records (Y-axis)

Distinct clustering patterns were identified for samples from various CHD subtypes in both the hydrophilic and hydrophobic metabolic profiling (Figure S3A, B and C). Table S2 showed all enrichment analysis findings. CHD-IAS could not be reliably distinguished from other groups (AUC, 0.607; P-value, 0.46) based on the hydrophilic metabolomics, while CHD-CMP also showed poor distinction from other groups (AUC, 0.53; P-value, 0.83) based on the hydrophobic metabolomics. The Arachidonic acid metabolism [7, 8] and Monoacylglycerols pathways are consistently and significantly enriched in all three CHD subtypes (Figure S3D and E). Additionally, the Linoleic acid metabolism [9, 10], serotonergic synapse, and spingoid bases pathways are significantly enriched solely in the IAS and TOF subtypes of CHD. Moreover, Quinones and hydroquinones [11] were found to be significantly enriched only in CHD-CMP, while Arginine and ornithine metabolism showed significant enrichment exclusively in CHD-IAS. These findings suggest the presence of diverse metabolic pathway changes among the different CHD subtypes, emphasizing the importance of considering both hydrophilic and hydrophobic metabolites when constructing a CHD diagnosis panel.

Comparing CHD patients with healthy controls, we identified 3 biomarker metabolites (P-value < 0.05) through univariate analysis (Table S3), namely PC(d16:1–22:3), C14:1-Carnitine, and C12-Carnitine (Fig. 2A and **B**). The logistic model exhibited high accuracy in distinguishing between CHD patients and healthy controls, achieving an AUC of 0.982 (95% CI: 0.92-1.00) (Fig. 2C). For CHD subtyping by the LightGBM model, there are 12 crucial metabolites required to achieve 80% cumulative importance (Fig. 2D). These significant metabolites include Alanine, C10-Carnitine, TG(16:0–16:0–20:2), TG(16:1–18:2–18:3), PE(a20:0–20:2), C10:1-Carnitine, Octacosanoic acid(28:0), C14:1OH-Carnitine, C8:1-Carnitine, Asparagine, C0-Carnitine, and d18:0 CE. OPLS-DA analysis was carried out using the aforementioned 12 metabolites (Fig. 2E). Figure S4 revealed the AUCs for distinguishing CMP vs. Other, IAS vs. Other, and TOF vs. Other. These findings indicate promising discriminatory capabilities of the selected metabolites in identifying different CHD subtypes when compared to the other groups.

**Fig. 2:**
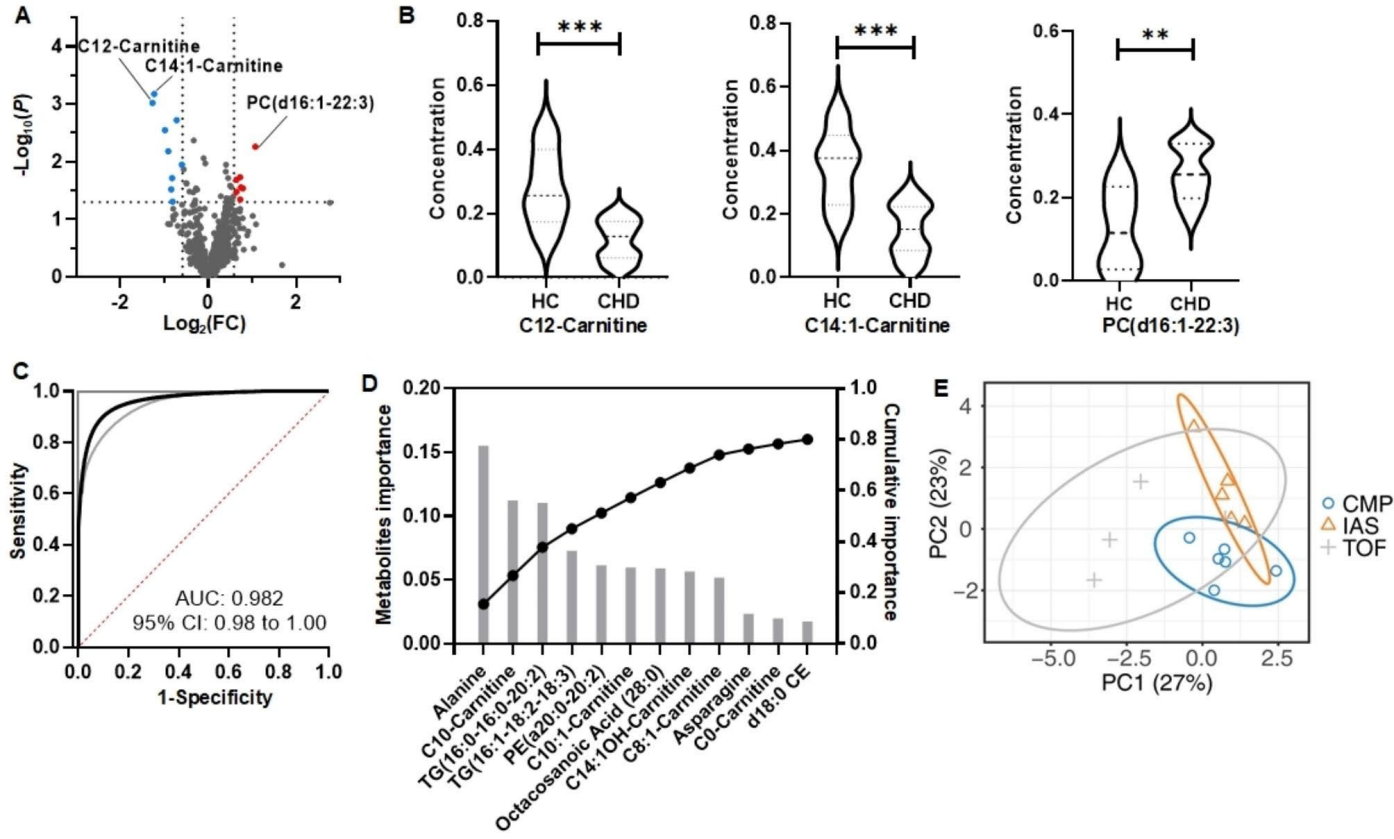
The application of targeted metabolomics to discover CHD diagnosis and subtyping biomarker metabolites using DBS samples. (**A**) Volcano plots for screening significant changed metabolites associated with CHD, the metabolites with P value < 0.05 and fold change > 1.5 are marked as red dots and the metabolites with P value < 0.05 and fold change < 0.67 are marked as blue dots. (**B**) violin plots for three biomarker metabolites for CHD diagnosis. *: Student’s P value < 0.05, **: P value < 0.01, ***: P value < 0.001. (**C**) the smoothed receiver operating characteristic curve (AUC ROC) of logistic model based on three biomarkers. 95% confidence intervals are shown in grey lines. (**D**) the importance score of the 12 metabolites associated with CHD subtyping which have an 80% cumulative importance in total. (**E**) PLS-DA cluster results using 12 metabolites for CHD subtyping

The study results underline the feasibility of using long-term stored DBS to identify metabolic biomarker panel for early CHD detection and assessment. It provides the basis for the future investigation of large-scale clinical trial for DBS biomarker panel as a molecular surrogate to assess congenital cardiac abnormalities. Moreover, by further investigating the biomarker metabolites and their underlying enriched pathways, we may gain deeper insights into the mechanisms underlying CHD pathophysiology. With better understanding of CHD development, there are implications for future research, treatments, and improved patient outcomes.

### Electronic supplementary material

Below is the link to the electronic supplementary material.

Supplementary Material 1: Additional file 1: **Supplementary Methods**. **Figure S1**. Study workflow diagram to apply metabolomic analytics to the neonate DBS samples and to discover CHD biomarkers. Abbreviations: CHD- Congenital heart disease, TOF- Tetralogy of Fallot, IAS- Inherited arrhythmias syndromes, CMP- Cardiomyopathies. **Figure S2.** Statistical distribution of DBS Samples storage times in California Department of Public Health Lab. **Figure S3.** Orthogonal partial least squares discriminant analysis (OPLS-DA) using the global hydrophilic and hydrophobic metabolic pro-filing results of health control (HC), CHD-Tetralogy of Fallot (TOF), CHD-inherited arrhythmia syndromes (IAS) and CHD-cardiomyopathies (CMP). (A) clustering results of hydrophilic metabolic profiling (B) clustering results of hydrophobic met-abolic profiling. (C) AUC and P value for clustering each subtype from other groups. Significant Metabolic pathways altered in different CHD subtypes. Pathway enrichment analysis on the (D) global hydrophilic and (E) hydrophobic metabolic profiling. All significant changed components (P value < 0.05, Student’s t-test) in CHD-Tetralogy of Fallot (TOF), CHD-inherited arrhythmia syndromes (IAS) and CHD-cardiomyopathies (CMP) are mapping to KEGG metabolic pathways and Lipid Map database, re-spectively. *: P value < 0.05, **: P value < 0.01, ***: P value < 0.001. **Figure S4.** CHD Subtyping modeling with targeted metabolomic profiling analysis of newborn DBS samples. (A) Confusion matrix. (B) AUC curves to demonstrate the performance to diagnose CHD subtypes. **Table S1**. The demographics of CDPH DBS Samples. **Table S2.** Global metabolites enrichment analysis. **Table S3.** Univariate analysis result of target metabolism

## Data Availability

The datasets used or analyzed during the current study are available from the corresponding author on reasonable request due to privacy.

## Abbreviations

AUC: The area under the receiver operating characteristic curve
CDPH: California Department of Public Health
CE: Cholesterol esters
CHD: Congenital heart disease
CMP: 3 dilated, 3 hypertrophic cardiomyopathy
DBS: Dried blood spot
IAS: 2 Brugada, 3 Long QT syndrome
LC-MS/MS: Liquid chromatography with tandem mass spectrometry
LC-MS: Liquid chromatography-mass spectrometry
OPLS-DA: Orthogonal partial least squares discriminant analysis
PC: Phosphatidylcholine
TG: Triglyceride
TOF: Tetralogy of Fallot
