# Correction: Exploring the feasibility of using long-term stored newborn dried blood spots to identify metabolic features for congenital heart disease screening

**DOI:** 10.1186/s40364-023-00546-w

**Published:** 2023-11-22

**Authors:** Scott R. Ceresnak, Yaqi Zhang, Xuefeng B. Ling, Kuo Jung Su, Qiming Tang, Bo Jin, James Schilling, C. James Chou, Zhi Han, Brendan J. Floyd, John C. Whitin, Kuo Yuan Hwa, Karl G. Sylvester, Henry Chubb, Ruben Y. Luo, Lu Tian, Harvey J. Cohen, Doff B. McElhinney

**Affiliations:** 1 Department of Pediatrics, Stanford University School of Medicine, Stanford, CA 94305 USA; 2 College of Automation, Guangdong Polytechnic Normal University, 293 Zhongshan Avenue West, Tianhe District, Guangzhou, 510665 China; 3 Department of Surgery, Stanford University School of Medicine, Stanford, CA 94305 USA; 4 mProbe Inc, Palo Alto, CA 94303 USA; 5 The Center for Biomedical Industries, National Taipei University of Technology, Taipei, Taiwan; 6 Department of Pathology, Stanford University School of Medicine, Stanford, CA 94305 USA; 7 Department of Biomedical Data Science, Stanford University School of Medicine, Stanford, CA 94305 USA; 8 Departments of Cardiothoracic Surgery, Stanford University School of Medicine, Stanford, CA 94305 USA

**Biomarker Research (2023) 11:97**

10.1186/s40364-023-00536-y

The original article [1] contained an error in the affiliations of co-authors, Yaqi Zhang and Xuefeng B. Ling due to a no-fault misinterpretation during the proofing process. The affiliations have since been amended to reflect the affiliations that were originally requested by the authors.

