# Supplementary material for: Exploring the feasibility of using long-term stored newborn dried blood spots to identify metabolic features for congenital heart disease screening
Source: Biomark Res. 2023 Nov 13;11:97. doi: 10.1186/s40364-023-00536-y (PMC10644604; doi:10.1186/s40364-023-00536-y)
Supplement: Supplementary file 1 — Supplementary Material 1: Additional file 1: Supplementary Methods. Figure S1. Study workflow diagram to apply metabolomic analytics to the neonate DBS samples and to discover CHD biomarkers. Abbreviations: CHD- Congenital heart disease, TOF- Tetralogy of Fallot, IAS- Inherited arrhythmias syndromes, CMP- Cardiomyopathies. Figure S2. Statistical distribution of DBS Samples storage times in California Department of Public Health Lab. Figure S3. Orthogonal partial least squares discriminant analysis (OPLS-DA) using the global hydrophilic and hydrophobic metabolic pro-filing results of health control (HC), CHD-Tetralogy of Fallot (TOF), CHD-inherited arrhythmia syndromes (IAS) and CHD-cardiomyopathies (CMP). (A) clustering results of hydrophilic metabolic profiling (B) clustering results of hydrophobic met-abolic profiling. (C) AUC and P value for clustering each subtype from other groups. Significant Metabolic pathways altered in different CHD subtypes. Pathway enrichment analysis on the (D) global hydrophilic and (E) hydrophobic metabolic profiling. All significant changed components (P value < 0.05, Student’s t-test) in CHD-Tetralogy of Fallot (TOF), CHD-inherited arrhythmia syndromes (IAS) and CHD-cardiomyopathies (CMP) are mapping to KEGG metabolic pathways and Lipid Map database, re-spectively. *: P value < 0.05, **: P value < 0.01, ***: P value < 0.001. Figure S4. CHD Subtyping modeling with targeted metabolomic profiling analysis of newborn DBS samples. (A) Confusion matrix. (B) AUC curves to demonstrate the performance to diagnose CHD subtypes. Table S1. The demographics of CDPH DBS Samples. Table S2. Global metabolites enrichment analysis. Table S3. Univariate analysis result of target metabolism [file 40364_2023_536_MOESM1_ESM.docx]

**Supplementary Materials for**

**Exploring the Feasibility of Using Newborn Dried Blood Spots to Identify Metabolic Features for Congenital Heart Disease Screening**

Scott R. Ceresnak ^1^*, Yaqi Zhang ^2,3^*, Xuefeng B. Ling ^3^, Kuo Jung Su ^4^, Qiming Tang ^4^, Bo Jin ^4^, James Schilling ^4^, C. James Chou ^3^, Zhi Han ^3^, Brendan J. Floyd ^5^, John C. Whitin ^5^, Kuo Yuan Hwa ^6^, Karl G Sylvester ^3^, Henry Chubb ^1^, Ruben Y. Luo ^7^, Lu Tian ^8^, Harvey J. Cohen ^5^, Doff B. McElhinney ^1^

^1^ Departments of Cardiothoracic Surgery, Stanford University School of Medicine, Stanford, CA 94305, USA;

^2^ College of Automation, Guangdong Polytechnic Normal University, Guangzhou 510665, China;

^3^ Department of Surgery, Stanford University School of Medicine, Stanford, CA 94305, USA;

^4^ mProbe Inc., Palo Alto, CA 94303, USA;

^5^ Department of Pediatrics, Stanford University School of Medicine, Stanford, CA 94305, USA;

^6^ The Center for Biomedical Industries, National Taipei University of Technology, Taipei, Taiwan;

^7^ Department of Pathology, Stanford University School of Medicine, Stanford, CA 94305, USA;

^8^ Department of Biomedical Data Science, Stanford University School of Medicine, Stanford, CA 94305, USA.

***** Correspondence: ceresnak@stanford.edu (Falk Building 870 Quarry Rd Extension Palo Alto, CA 94304)

yaqizhang@gpnu.edu.cn (293 Zhongshan Avenue West, Tianhe District, Guangzhou 510665)

**This file includes:**

Supplementary Methods

Supplementary Figures. S1 to S4

Supplementary Tables. S1 to S3

Supplementary Methods

1. Ethics statement

This method development study involving human participants was reviewed and approved by ethics committees at Stanford University.

2. Study design

The workflow for this study, as depicted in **Figure S1**, was based on the collected dried blood spot (DBS) samples. Both global hydrophilic/hydrophobic and targeted liquid chromatography-tandem mass spectrometry (LC-MS/MS) metabolomic assays were conducted, adhering to the newborn screening (NBS) DBS processing method. To verify the reliability of the metabolic profiling from samples that had been stored for years, a correlation analysis was carried out comparing the absolutely-quantified metabolite concentration with the California Department of Public Health (CDPH) gold standard. We identified significant metabolic pathways and features related to CHD and its subtypes. Finally, models for CHD diagnosis and subtyping were established to validate the effectiveness of the CHD-associated metabolic biomarker panel within the DBS samples.

3. Cohorts

We randomly assembled a retrospective cohort of DBS profiling datasets from the CDPH Biobank. These DBS samples had been stored for up to 15 years since the time of newborn testing analysis, consented to be preserved at -20°C in the CDPH Biobank. In total, we included 20 clinical DBS samples, consisting of 5 healthy controls (HC) and 15 cases of CHD spanning different categories. The 15 CHD cases included 4 instances of Tetralogy of Fallot (TOF), 5 cases of inherited arrhythmia syndromes (IAS), and 6 instances of cardiomyopathies (CMP). **Table S1** summarizes the cohort demographics and clinical characteristics with Fisher's exact test.

4. Sample preparation

The DBS samples were retrieved from -20°C freezers and thawed on ice. Each DBS sample was cut into 3mm diameter sections, and two of these sections were transferred into the same Eppendorf tube.

4.1. Global hydrophilic and targeted metabolomics

We followed our previously developed standard operational protocols [1] with slight modifications of global hydrophilic and targeted metabolomics in this study, including sample preprocessing, mass spectrometry signal acquisition, quality control (QC), data pre-processing, and metabolite biomarker structural identification. In three kinds of omics dataset, we retained only those features with a missing data ratio (NA ratio) of less than 20%. For handling missing values, k-nearest neighbors imputation with a k-value of 3 were implemented. For untargeted metabolism (global metabolites and global lipids), the method of Median over Median (MOM) normalization with QC samples as baseline was employed.

For global hydrophilic metabolomics, a mixture of 250 μL extraction buffer (pre-chilled to -20 °C) consisting of methanol, acetonitrile and ddH2O (5:3:2 v/v) was added to each tube containing DBS punches. Samples were vortexed and centrifuged at 10,000 g for 10 min at 4°C. Supernatant (180 μL) from each sample was transferred into a clean Eppendorf tube. 100 µL of each sample extract was transfer into an auto-sampler vial for UHPLC-MS (Ultra-High Performance Liquid Chromatography) analysis. The mobile phase A was water with 0.1% formic acid in water, and mobile phase B was acetonitrile with 0.1% formic acid. The separation was carried out using isocratic elution with 80% B at a flow rate of 0.05 ml/min for a total run time of 3 min. The eluted metabolites were detected by a Q Exactive Plus mass spectrometer (Thermo Fisher) operated in full scan setup using both electrospray positive and negative modes, operating separately as two independent runs. The conditions of ionization source were set at 4 kV for spray voltage, 15 for sheath gas, 12 for aux gas, 325°C for capillary temperature, 55 for S-lens, and 250°C for vaporizer temperature. The MS spectra were acquired with 2 µscans using an AGC target of 1e6 and a resolution of 140,000 (FWHM at 200 m/z) from 60 to 900 m/z. The column oven was maintained at 25°C throughout the analysis.

For targeted metabolomics, a mixture of 200 µL methanol / acetonitrile (1:1, v/v) was added into the tube containing DBS punches. Samples were vortexed vigorously and centrifuged at 10,000 g for 10 min at 4°C. The supernatant of each sample was transferred into a new tube and dried under nitrogen stream. 10 µL of Internal Standard Solution, 90 µL of Extraction Buffer, and 200 µL hexane were added into the reconstituted tube for extraction. The sample was vortexed vigorously for 1 min and centrifuged at 12,000 g for 5 min. 180 µL of upper layer was transferred into another 1.5-mL centrifugal tube and dried under nitrogen stream again. The residue was reconstituted with 100 µL of Derivatization Buffer. The reconstituted sample was incubated at 95°C for 15 min. After derivatization, 100 µL of each reconstituted sample was transferred into an auto-sampler vial for the analysis of fatty acids. 80 µL of lower layer was transferred into an auto-sampler vial for the analysis of amino acids and acylcarnitines. The mobile phase A was water with 0.5% formic acid and 10 mM Ammonium Formate, mobile phase B was methanol with 0.5% formic acid and10 mM Ammonium Formate. The separation was carried out using isocratic elution with 50% B at a flow rate of 0.30 ml/min. The eluted metabolites were detected by an Altis mass spectrometer (Thermo Fisher) operated in SRM setup using electrospray positive mode. The conditions of ionization source were set at 3.5 kV for spray voltage, 20 for sheath gas, 5 for aux gas, 300°C for ion transfer tube temperature, 200°C for vaporizer temperature. The MS spectra were acquired using an cycle time of 0.8 and Q1 resolution (FWHM) of 0.7, Q3 resolution (FWHM) of 0.7, CID gas of 1.5, chromatographic peak width of 12. The column oven was maintained at 30°C throughout the analysis.

4.2. Global hydrophobic metabolomics

For global hydrophobic metabolomics, a mixture of 400 μL of chloroform / methanol (1:1, v/v) and 200 µL of water with lithium chloride were added to each tube containing DBS punches. Afterwards, the sample was vortexed rigorously for 30 sec and centrifuged at 12,000 g for 5 min. The bottom layer of each sample was transfered into another tube. The top layer was re-extracted with 400 µL of chloroform and vortexed for 30 sec and centrifuged at 12,000 g for 5 min. The bottom layer was removed and combined with extract from previous trial. The combined extract was dried under nitrogen and reconstituted with 100 µL of methanol: chloroform (1:1, v/v). Thereafter, the DBS extract was transferred into auto-sampler vial with micro-insert for LC/MS analysis.

For global hydrophobic metabolomics, 5 μL of DBS extract was injected via a Vanquish UHPLC system. The eluted metabolites were detected by a Q Exactive Plus mass spectrometer (Thermo Fisher) operated in full scan setup using both electrospray positive and negative modes, operating separately as two independent runs. The mobile phase was methanol with 10 mM ammonium acetate at a flow rate of 0.1 mL/min for a total run time of 3 minutes. The conditions of ionization source were set at 3.2 kV for spray voltage, 20 for sheath gas, 5 for aux gas, 300°C for capillary temperature, 55 for S-lens, and 250°C for vaporizer temperature. The MS spectra were acquired with 2 scans using an AGC target of 1e6 and a resolution of 120,000 (FWHM at 200 m/z) from 200 to 1200 m/z. The column oven was maintained at 25°C throughout the analysis.

5. Metabolic pathway enrichment analysis with global hydrophilic and hydrophobic metabolomics

To carry out pathway enrichment analysis on both hydrophilic and hydrophobic mass spectrometric profiling results, a univariate analysis was utilized to compute the fold change and P-value (using Student's t-test) for each component across the three different CHD subtypes. Following the correction for false discovery, components that showed significant changes (adjusted P-value < 0.05) were chosen for subsequent analysis.

All significantly altered hydrophilic metabolites were categorized into KEGG pathways [2] for further examination, while significantly changed hydrophobic metabolites were grouped into the Lipid Map database [3]. Enriched pathways of significance were identified with a Fisher's exact test P-value of < 0.05.

6. Statistic learning for multi-class classification

Orthogonal partial least squares discriminant analysis (OPLS-DA) was performed using global hydrophilic and hydrophobic metabolic profiling results. Unsupervising clustering results were used to visualize the two-dimensional clustering patterns of HC, TOF, IAS and CMP DBS samples. With OPLS-DA results, the performance of the discrimination between two of these DBS sample categories was assessed using a receiver operating characteristic curve (ROC) and the area under the curve (AUC).

To distinguish between CHD patients and healthy controls, a logistic model [4] was constructed based on targeted metabolomic profiling results. For CHD subtyping, the importance of identified targeted metabolites was determined using a gradient boosting machine provided by the LightGBM library [5]. Analysis was implemented with default parameters, and the metric used for early stopping was set to the error rate for multi-class classification [6]. Metabolites were then ranked based on their normalized importance scores. Metabolites with cumulative importance greater than 80% were selected as biomarkers.

The significantly relevant biomarker metabolites associated with CHD diagnosis and subtyping were then proposed and discussed.

All statistical analyses were conducted using various R packages, providing a robust and comprehensive framework for analyzing and interpreting the metabolomics data in this study. The "pamr" package was employed to perform OPLS-DA analysis. To determine feature importance, we utilized the "LightGBM" package. A LightGBM model was trained on our dataset, and the feature importance of each metabolite in this fitted model were exported. For data visualization, we relied on GraphPad Prism.

Supplementary Figures. S1 to S4


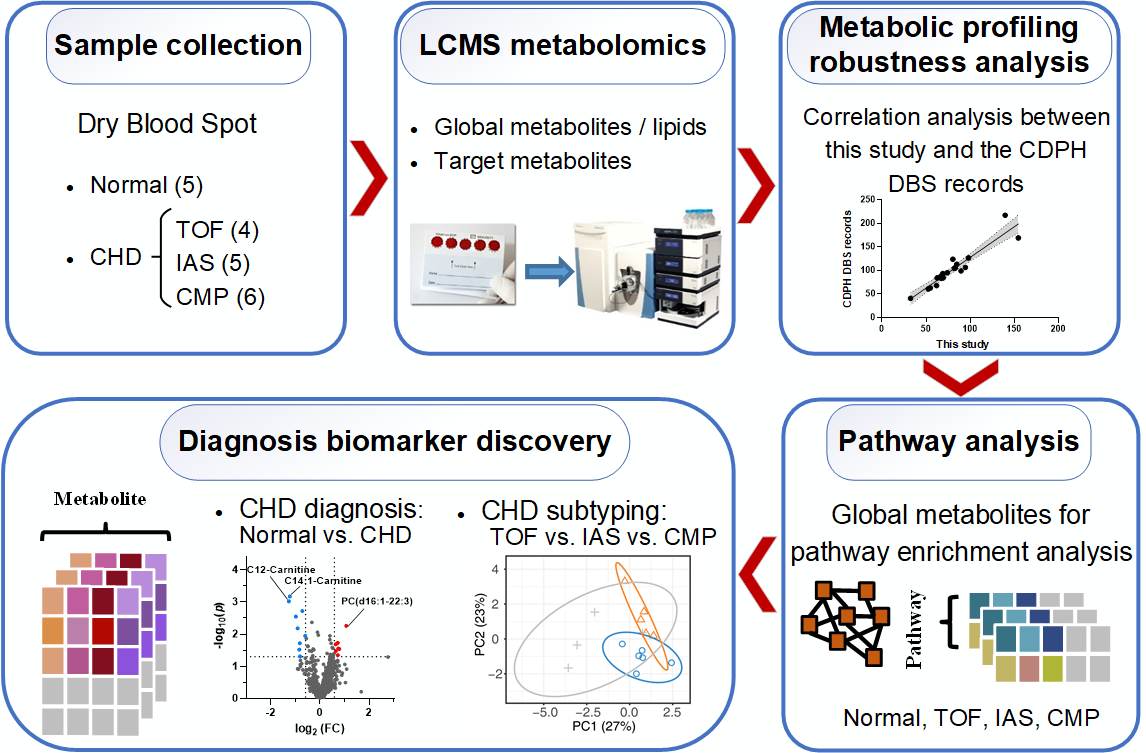


**Figure S1.** Study workflow diagram to apply metabolomic analytics to the neonate DBS samples and to discover CHD biomarkers. Abbreviations: CHD- Congenital heart disease, TOF- Tetralogy of Fallot, IAS- Inherited arrhythmias syndromes, CMP- Cardiomyopathies.


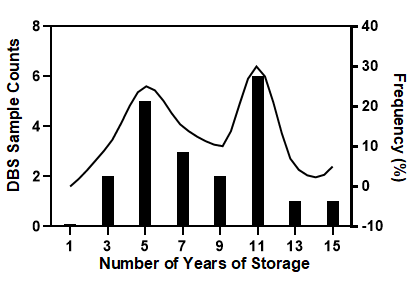


**Figure S2.** Statistical distribution of DBS Samples storage times in California Department of Public Health Lab.


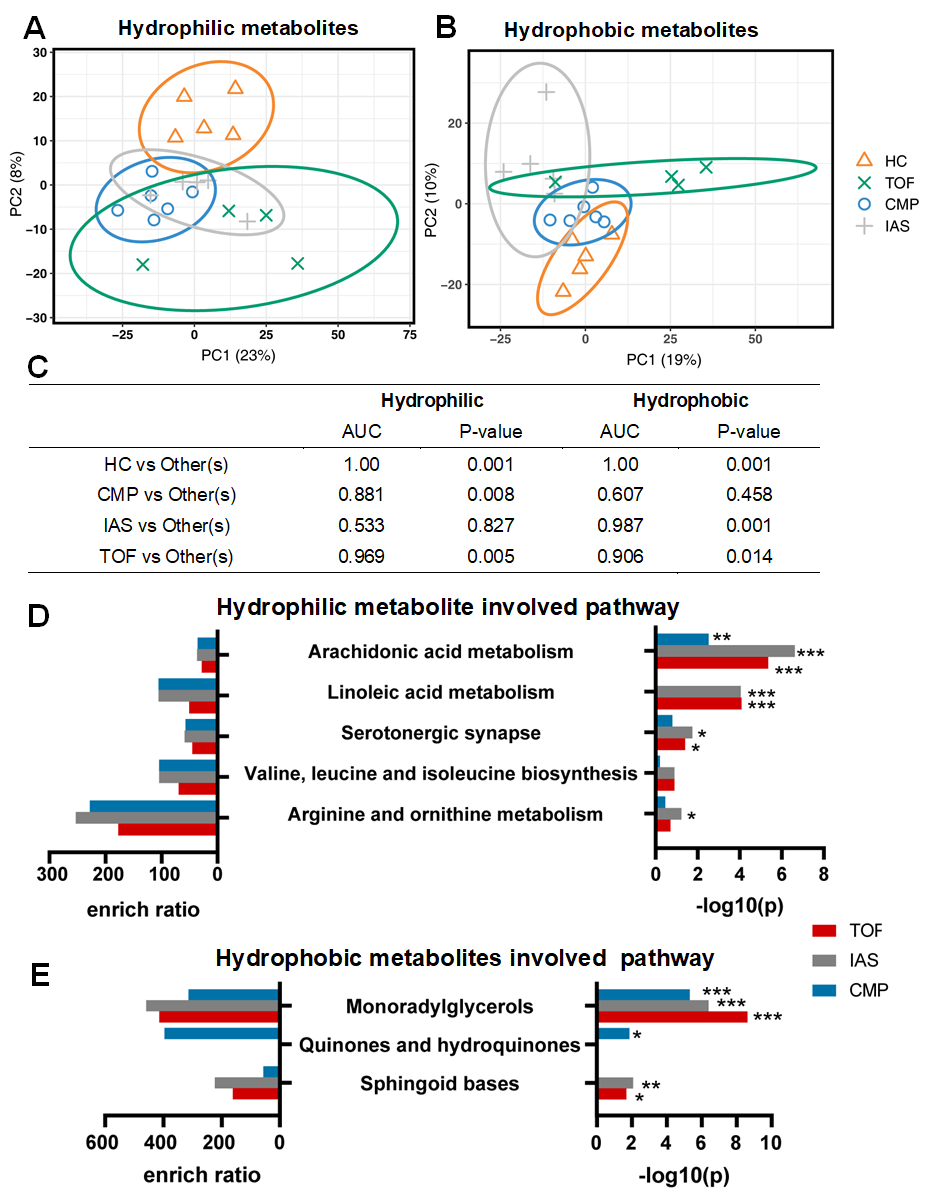


**Figure S3****.** Orthogonal partial least squares discriminant analysis (OPLS-DA) using the global hydrophilic and hydrophobic metabolic profiling results of health control (HC), CHD-Tetralogy of Fallot (TOF), CHD-inherited arrhythmia syndromes (IAS) and CHD-cardiomyopathies (CMP). A) clustering results of hydrophilic metabolic profiling B) clustering results of hydrophobic metabolic profiling. C) AUC and P value for clustering each subtype from other groups. Significant Metabolic pathways altered in different CHD subtypes. Pathway enrichment analysis on the D) global hydrophilic and E) hydrophobic metabolic profiling. All significant changed components (P value <0.05, Student’s t-test) in CHD-Tetralogy of Fallot (TOF), CHD-inherited arrhythmia syndromes (IAS) and CHD-cardiomyopathies (CMP) are mapping to KEGG metabolic pathways and Lipid Map database, respectively. *: P value <0.05, **: P value <0.01, ***: P value <0.001.


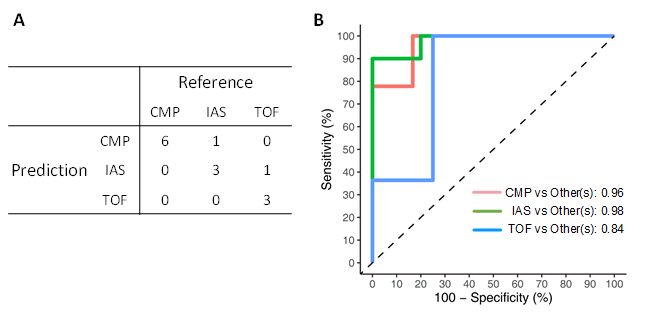


**Figure S4.** CHD Subtyping modeling with targeted metabolomic profiling analysis of newborn DBS samples. (A) Confusion matrix. (B) AUC curves to demonstrate the performance to diagnose CHD subtypes.

Supplementary Tables. S1 to S3

**Table S1.** The demographics of CDPH DBS Samples.

| **Characters** | **Control** | **CHD** | | | **P-value** |
| --- | --- | --- | --- | --- | --- |
|  |  | TOF | IAS | CMP |  |
| **Total** | 5 | 4 | 5 | 6 |  |
| **Diagnosis** |  |  |  |  | < 0.001 |
| Normal | 5 (100) | 0 (0) | 0 (0) | 0 (0) |  |
| Tetralogy of Fallot | 0 (0) | 4 (100) | 0 (0) | 0 (0) |  |
| Brugada syndrome | 0 (0) | 0 (0) | 2 (40) | 0 (0) |  |
| Long QT syndrome | 0 (0) | 0 (0) | 3 (60) | 0 (0) |  |
| Dilated cardiomyopathy | 0 (0) | 0 (0) | 0 (0) | 3 (50) |  |
| Hypertrophic cardiomyopathy | 0 (0) | 0 (0) | 0 (0) | 3 (50) |  |
|  |  |  |  |  |  |
| **Gender** |  |  |  |  | 0.93 |
| Female | 2 (40) | 2 (50) | 3 (60) | 2 (33.3) |  |
| Male | 3 (60) | 2 (50) | 2 (40) | 4 (66.7) |  |
|  |  |  |  |  |  |
| **Race** |  |  |  |  | 0.17 |
| Asian | 0 (0) | 1 (25) | 0 (0) | 1 (16.7) |  |
| Black | 1 (20) | 0 (0) | 0 (0) | 0 (0) |  |
| Hispanic | 0 (0) | 1 (25) | 4 (80) | 3 (50) |  |
| White | 4 (80) | 2 (50) | 1 (20) | 2 (33.3) |  |
|  |  |  |  |  |  |
| **CDPH DBS NBS records** |  |  |  |  |  |
| Thyroid stimulating hormone, mean (SD) | 6.7 (4.7) | 5.6 (5.4) | 5.5 (3.9) | 4.4 (1.7) | 0.82 |
| C8-Carnitine, mean (SD) | 0.1 (0) | 0 (0) | 0.1 (0) | 0.1 (0) | 0.011 |
| Galactose-1-phosphate uridyl transferase, median (IQR) | 247.2 (217,267.5) | 219.1 (170.6,274.6) | 269.5 (260.4,271) | 308.5 (304.3,314.1) | 0.17 |
| Valine, mean (SD) | 105.9 (19.3) | 102.7 (60.9) | 114.3 (40.7) | 90 (20.9) | 0.74 |
| C18:1-Carnitine, mean (SD) | 1.2 (0.3) | 0.9 (0.5) | 1 (0.3) | 1.2 (0.3) | 0.51 |
| C160H-Carnitine /C16-Carnitine Ratio, mean (SD) | 0 (0) | 0 (0) | 0 (0) | 0 (0) | 0.16 |
| C16:1-Carnitine, mean (SD) | 0.3 (0) | 0.1 (0.1) | 0.2 (0.1) | 0.3 (0.1) | 0.020 |
| 17-Hydroxyprogesterone (17-OHP), mean (SD) | 13.3 (8.9) | 18.3 (8.8) | 22.3 (18.6) | 10.9 (3.4) | 0.38 |
| C3-Carnitine / C2-Carnitine Ratio, mean (SD) | 0.1 (0) | 0.1 (0) | 0.1 (0) | 0.1 (0) | 0.29 |
| C14-Carnitine, mean (SD) | 0.3 (0) | 0.2 (0.1) | 0.2 (0) | 0.2 (0.1) | 0.005 |
| Methionine, mean (SD) | 28 (6.1) | 25.5 (12.4) | 20.2 (4.7) | 26 (5.5) | 0.40 |
| Alanine, mean (SD) | 230 (96.8) | 369.8 (180.5) | 307.2 (74) | 307.3 (109) | 0.38 |
| Immunoreactive trypsinogen, mean (SD) | 17.7 (7.7) | 15.9 (2.5) | 30.8 (16.1) | 18.6 (8.3) | 0.13 |
| Ornithine/Citrulline Ratio, median (IQR) | 5.7 (4.7,7.5) | 6.5 (5.5,8.8) | 4.8 (4.8,8.4) | 4.6 (4.3,4.9) | 0.39 |
| Arginine/Ornithine Ratio, mean (SD) | 0.1 (0) | 0.1 (0.1) | 0.1 (0.1) | 0.1 (0.1) | 0.92 |
| Leucine/Isoleucine, median (IQR) | 99.1 (93.4,113.2) | 86.7 (61.2,133.5) | 95.4 (62.8,106.5) | 88.9 (85.2,100.7) | 0.84 |
| C12-Carnitine, mean (SD) | 0.2 (0.1) | 0 (0) | 0.1 (0.1) | 0.1 (0) | 0.016 |
| C18:2-Carnitine, median (IQR) | 0.1 (0.1,0.2) | 0.3 (0.2,0.6) | 0.1 (0.1,0.3) | 0.2 (0.1,0.2) | 0.28 |
| Glycine, mean (SD) | 525.6 (89.3) | 448 (213.7) | 471.8 (105.1) | 553.5 (68.6) | 0.52 |
| C6-Carnitine, mean (SD) | 0.1 (0) | 0 (0) | 0 (0) | 0.1 (0) | 0.097 |
| C5OH-Carnitine, median (IQR) | 0.2 (0.1,0.2) | 0.2 (0.2,0.2) | 0.2 (0.2,0.3) | 0.2 (0.2,0.2) | 0.58 |
| Succinylacetone, mean (SD) | 0.5 (0.3) | 0.2 (0.1) | 0.6 (0.4) | 0.4 (0.3) | 0.33 |
| C12:1-Carnitine, mean (SD) | 0.1 (0.1) | 0 (0) | 0.1 (0.1) | 0.1 (0) | 0.018 |
| Arginine | 8.6 (2.6) | 8.8 (7.9) | 9.2 (3.1) | 7.5 (6.6) | 0.96 |
| C18:1OH-Carnitine, mean (SD) | 0 (0) | 0 (0) | 0 (0) | 0 (0) | 0.18 |
| C3DC-Carnitine /C10-Carnitine Ratio, mean (SD) | 1.3 (0.4) | 2.7 (0.9) | 1.7 (0.6) | 2.1 (1.2) | 0.16 |
| FC, mean (SD) | 19.6 (5.9) | 28 (11.2) | 25.3 (12.5) | 19 (4.6) | 0.35 |
| Leucine/Alanine Ratio, mean (SD) | 0.5 (0.2) | 0.3 (0.1) | 0.3 (0.2) | 0.3 (0.1) | 0.14 |
| Citrulline/Arginine Ratio, mean (SD) | 2.1 (0.9) | 2.8 (2.2) | 1.7 (1) | 4.1 (3.4) | 0.33 |
| C14:2-Carnitine, mean (SD) | 0 (0) | 0 (0) | 0 (0) | 0 (0) | 0.1 |
| C4-Carnitine, median (IQR) | 0.3 (0.2,0.3) | 0.2 (0.1,0.3) | 0.2 (0.2,0.3) | 0.2 (0.2,0.2) | 0.69 |
| C8:1-Carnitine, mean (SD) | 0.1 (0) | 0.1 (0) | 0.1 (0) | 0.1 (0) | 0.22 |
| Phenylalanine/Tyrosine Ratio, median (IQR) | 0.6 (0.6,0.7) | 0.9 (0.9,1) | 0.7 (0.7,0.8) | 0.6 (0.6,0.7) | 0.036 |
| C2-Carnitine, mean (SD) | 23.7 (9.8) | 26.6 (11.4) | 20 (4.5) | 23.6 (7.2) | 0.70 |
| Biotinidase, mean (SD) | 43.4 (10.3) | 41.7 (5.6) | 51.4 (13.8) | 42.7 (7.5) | 0.42 |
| C5DC-Carnitine, mean (SD) | 0.2 (0.1) | 0.1 (0) | 0.2 (0.1) | 0.2 (0.1) | 0.10 |
| C18OH-Carnitine, median (IQR) | 0 (0,0) | 0 (0,0) | 0 (0,0) | 0 (0,0) | 0.086 |
| C3-Carnitine, mean (SD) | 1.6 (0.4) | 2.1 (0.9) | 1.6 (0.5) | 2.2 (0.6) | 0.32 |
| C18-Carnitine, mean (SD) | 0.8 (0.2) | 0.8 (0.2) | 0.7 (0.1) | 0.8 (0.2) | 0.45 |
| C14:1-Carnitine / C12:1-Carnitine Ratio, mean (SD) | 1.3 (0.1) | 1.7 (0.9) | 1.2 (0.4) | 1.6 (0.4) | 0.51 |
| C10-Carnitine, mean (SD) | 0.1 (0) | 0 (0) | 0.1 (0) | 0.1 (0) | 0.005 |
| C5:1-Carnitine, median (IQR) | 0 (0,0) | 0 (0,0) | 0 (0,0) | 0 (0,0) | 0.51 |
| 5-Oxoproline, mean (SD) | 21.8 (5) | 32 (7.4) | 26.8 (6.6) | 25.2 (8.2) | 0.22 |
| C10:1-Carnitine, mean (SD) | 0.1 (0) | 0 (0) | 0.1 (0) | 0 (0) | 0.24 |
| C5-Carnitine, mean (SD) | 0.1 (0) | 0.1 (0.1) | 0.1 (0) | 0.1 (0) | 0.98 |
| Phenylalanine, mean (SD) | 63.9 (8.2) | 69.8 (33.3) | 55.4 (19.7) | 66.6 (15.8) | 0.72 |
| Proline, mean (SD) | 203.4 (38.2) | 159.8 (73.4) | 180.8 (43.6) | 179.2 (33.7) | 0.59 |
| C16OH-Carnitine, mean (SD) | 0 (0) | 0 (0) | 0 (0) | 0 (0) | 0.032 |
| Tyrosine, mean (SD) | 103.6 (17.7) | 72.6 (35.5) | 68.7 (30.1) | 102.7 (32.7) | 0.15 |
| Ornithine, mean (SD) | 98.6 (44.5) | 103.8 (92.5) | 102 (53.1) | 79.8 (28) | 0.88 |
| FC / (C16-Carnitine + C18:1-Carnitine) Ratio, median (IQR) | 4.3 (3.9,5) | 8.1 (7.3,9) | 6.7 (5,7.1) | 4.5 (3.7,5) | 0.037 |
| C16-Carnitine, mean (SD) | 3.4 (1) | 2.5 (0.9) | 2.3 (0.3) | 3.1 (0.8) | 0.17 |
| C14OH-Carnitine, mean (SD) | 0 (0) | 0 (0) | 0 (0) | 0 (0) | 0.012 |
| C3DC-Carnitine, mean (SD) | 0.1 (0) | 0.1 (0) | 0.1 (0) | 0.1 (0) | 0.020 |
| C14:1-Carnitine, mean (SD) | 0.2 (0.1) | 0 (0) | 0.1 (0) | 0.1 (0) | 0.004 |
| C5-Carnitine /C3-Carnitine Ratio, mean (SD) | 0.1 (0) | 0.1 (0) | 0.1 (0) | 0.1 (0) | 0.28 |
| C8-Carnitine /C10-Carnitine Ratio, mean (SD) | 0.7 (0.1) | 1 (0.5) | 1 (0.3) | 0.8 (0.1) | 0.47 |
| Citrulline, mean (SD) | 16.6 (3.6) | 11.8 (3.9) | 14 (6) | 17 (3.2) | 0.24 |
| Valine / Phenylalanine Ratio, mean (SD) | 1.7 (0.3) | 1.5 (0.4) | 1.9 (0.4) | 1.4 (0.3) | 0.15 |

Values are mean ± SD or numbers (percentages). SD: Standard deviation; IQR: Interquartile Range. CHD: Congenital heart disease. TOF: Tetralogy of Fallot. IAS: Inherited arrhythmias syndromes. CMP: Cardiomyopathies.

**Table S2.** Global metabolites enrichment analysis

| **Pathway** | **Health vs. TOF** | | **Health vs. IAS** | | **Health vs. CMP** | |
| --- | --- | --- | --- | --- | --- | --- |
|  | **P- value** | **Enrich ratio** | **P-value** | **Enrich ratio** | **P-value** | **Enrich ratio** |
| **Hydrophilic metabolite involved pathway** |  |  |  |  |  |  |
| Arachidonic acid metabolism | 0.003 | 28.35 | 0.000 | 37.08 | 0.000 | 35.99 |
| Serotonergic synapse | 0.162 | 45.21 | 0.018 | 59.11 | 0.040 | 57.38 |
| D-Arginine and D-ornithine metabolism | 0.349 | 177.43 | 0.060 | 253.47 | 0.196 | 228.12 |
| Linoleic acid metabolism | 0.817 | 50.86 | 0.000 | 105.62 | 0.000 | 105.62 |
| Valine, leucine and isoleucine biosynthesis | 0.620 | 69.57 | 0.129 | 104.36 | 0.126 | 104.36 |
| Central carbon metabolism in cancer | 0.852 | 38.09 | 0.704 | 51.53 | 0.571 | 53.77 |
| alpha-Linolenic acid metabolism | 0.809 | 33.27 | 0.746 | 42.77 | 0.630 | 44.36 |
| Pentose and glucuronate interconversions | 1.000 | 16.22 | 0.638 | 35.49 | 0.630 | 35.49 |
| Ovarian steroidogenesis | 0.697 | 63.90 | 0.957 | 63.90 | 0.902 | 69.22 |
| Mineral absorption | 0.762 | 51.06 | 0.901 | 58.35 | 0.898 | 58.35 |
| Caffeine metabolism | 0.695 | 69.70 | 0.994 | 57.03 | 0.889 | 76.04 |
| African trypanosomiasis | 0.893 | 143.77 | 0.893 | 191.69 | 0.892 | 191.69 |
| Synthesis and degradation of ketone bodies | 0.917 | 170.39 | 0.882 | 255.58 | 0.880 | 255.58 |
| Vascular smooth muscle contraction | 0.842 | 83.86 | 0.932 | 95.84 | 0.931 | 95.84 |
| Prolactin signaling pathway | 0.792 | 126.74 | 0.988 | 101.39 | 0.948 | 126.74 |
| Protein digestion and absorption | 0.986 | 24.99 | 0.822 | 38.88 | 0.933 | 36.10 |
| Cocaine addiction | 0.956 | 125.18 | 0.944 | 187.78 | 0.943 | 187.78 |
| Alanine, aspartate and glutamate metabolism | 0.980 | 39.12 | 0.966 | 54.77 | 0.923 | 58.68 |
| GABAergic synapse | 0.937 | 113.59 | 0.989 | 113.59 | 0.945 | 151.46 |
| Type II diabetes mellitus | 0.917 | 170.39 | 0.977 | 170.39 | 0.977 | 170.39 |
| Ascorbate and aldarate metabolism | 0.970 | 25.55 | 0.969 | 33.21 | 0.968 | 33.21 |
| Alcoholism | 0.964 | 92.01 | 0.974 | 122.68 | 0.973 | 122.68 |
| D-Glutamine and D-glutamate metabolism | 0.972 | 72.59 | 0.997 | 72.59 | 0.952 | 108.89 |
| Amphetamine addiction | 0.988 | 75.73 | 0.989 | 113.59 | 0.988 | 113.59 |
| Butanoate metabolism | 1.000 | 17.39 | 0.984 | 36.51 | 0.984 | 36.51 |
| Phototransduction | 0.977 | 95.84 | 0.996 | 95.84 | 0.996 | 95.84 |
| Platelet activation | 0.984 | 62.59 | 0.994 | 78.24 | 0.994 | 78.24 |
| Galactose metabolism | 0.980 | 26.09 | 0.998 | 30.44 | 0.998 | 30.44 |
| C-type lectin receptor signaling pathway | 0.980 | 76.04 | 0.998 | 76.04 | 0.998 | 76.04 |
| Arginine biosynthesis | 0.998 | 34.79 | 0.990 | 57.98 | 0.989 | 57.98 |
| Oxytocin signaling pathway | 0.989 | 63.90 | 0.994 | 85.19 | 0.994 | 85.19 |
| Prostate cancer | 0.980 | 76.04 | 1.000 | 50.69 | 0.998 | 76.04 |
| Primary bile acid biosynthesis | 1.000 | 13.88 | 0.983 | 33.32 | 0.996 | 30.55 |
| Arginine and proline metabolism | 0.998 | 14.62 | 0.984 | 21.17 | 0.998 | 19.66 |
| Histidine metabolism | 0.999 | 20.83 | 0.992 | 31.93 | 0.992 | 31.93 |
| Necroptosis | 0.994 | 61.34 | 0.995 | 92.01 | 0.995 | 92.01 |
| Retinol metabolism | 0.997 | 34.35 | 0.991 | 53.98 | 0.997 | 49.07 |
| Taurine and hypotaurine metabolism | 0.987 | 44.36 | 1.000 | 44.36 | 1.000 | 44.36 |
| Dopaminergic synapse | 0.989 | 63.90 | 0.999 | 63.90 | 0.999 | 63.90 |
| Lysine degradation | 1.000 | 17.88 | 0.996 | 27.35 | 0.996 | 27.35 |
| Cushing syndrome | 0.994 | 54.44 | 1.000 | 54.44 | 1.000 | 36.30 |
| Nicotinate and nicotinamide metabolism | 0.999 | 18.25 | 1.000 | 18.25 | 0.994 | 27.37 |
| Regulation of lipolysis in adipocytes | 0.997 | 46.94 | 0.999 | 62.59 | 0.999 | 62.59 |
| Inflammatory mediator regulation of TRP channels | 0.997 | 27.54 | 0.999 | 35.05 | 0.999 | 35.05 |
| Pyruvate metabolism | 0.999 | 25.53 | 0.997 | 41.49 | 0.999 | 38.30 |
| Renin-angiotensin system | 0.997 | 50.69 | 1.000 | 50.69 | 1.000 | 50.69 |
| Citrate cycle (TCA cycle) | 0.997 | 38.34 | 1.000 | 46.01 | 1.000 | 46.01 |
| HIF-1 signaling pathway | 0.998 | 40.89 | 1.000 | 54.52 | 0.999 | 54.52 |
| Sphingolipid signaling pathway | 0.998 | 40.89 | 1.000 | 54.52 | 0.999 | 54.52 |
| Glycine, serine and threonine metabolism | 0.998 | 20.86 | 1.000 | 25.76 | 1.000 | 22.08 |
| Parkinson disease | 0.998 | 40.89 | 1.000 | 40.89 | 1.000 | 40.89 |
| Cortisol synthesis and secretion | 0.998 | 42.60 | 1.000 | 42.60 | 1.000 | 42.60 |
| Insulin secretion | 0.998 | 42.60 | 1.000 | 42.60 | 1.000 | 42.60 |
| Pantothenate and CoA biosynthesis | 0.999 | 27.38 | 1.000 | 39.12 | 1.000 | 39.12 |
| Oxidative phosphorylation | 0.999 | 35.94 | 1.000 | 47.92 | 1.000 | 47.92 |
| Carbohydrate digestion and absorption | 0.999 | 29.45 | 1.000 | 33.66 | 1.000 | 33.66 |
| Fructose and mannose metabolism | 0.999 | 18.93 | 1.000 | 21.04 | 1.000 | 21.04 |
| Insulin resistance | 0.999 | 33.98 | 1.000 | 33.98 | 1.000 | 33.98 |
| Phenylalanine metabolism | 0.999 | 17.04 | 1.000 | 21.30 | 1.000 | 22.15 |
| beta-Alanine metabolism | 1.000 | 20.97 | 1.000 | 32.95 | 1.000 | 35.94 |
| Glucagon signaling pathway | 1.000 | 27.22 | 1.000 | 36.30 | 1.000 | 36.30 |
| Proximal tubule bicarbonate reclamation | 1.000 | 31.84 | 1.000 | 31.84 | 1.000 | 42.45 |
| cAMP signaling pathway | 1.000 | 24.54 | 1.000 | 39.26 | 1.000 | 39.26 |
| Antifolate resistance | 1.000 | 31.84 | 1.000 | 21.22 | 1.000 | 21.22 |
| Sulfur metabolism | 1.000 | 22.53 | 1.000 | 33.80 | 1.000 | 30.98 |
| Retrograde endocannabinoid signaling | 1.000 | 25.49 | 1.000 | 42.48 | 1.000 | 42.48 |
| Phenylalanine, tyrosine and tryptophan biosynthesis | 1.000 | 22.53 | 1.000 | 27.54 | 1.000 | 30.04 |
| Selenocompound metabolism | 1.000 | 16.83 | 1.000 | 37.86 | 1.000 | 21.04 |
| Aldosterone synthesis and secretion | 1.000 | 25.35 | 1.000 | 31.68 | 1.000 | 31.68 |
| Shigellosis | 1.000 | 23.96 | 1.000 | 23.96 | 1.000 | 23.96 |
| Taste transduction | 1.000 | 20.97 | 1.000 | 26.96 | 1.000 | 26.96 |
| Pentose phosphate pathway | 1.000 | 20.03 | 1.000 | 25.04 | 1.000 | 27.54 |
| Tryptophan metabolism | 1.000 | 12.02 | 1.000 | 16.03 | 1.000 | 15.14 |
| Vitamin B6 metabolism | 1.000 | 19.56 | 1.000 | 31.30 | 1.000 | 31.30 |
| Renin secretion | 1.000 | 21.22 | 1.000 | 21.22 | 1.000 | 21.22 |
| Neuroactive ligand-receptor interaction | 1.000 | 15.88 | 1.000 | 19.28 | 1.000 | 18.15 |
| Nitrogen metabolism | 1.000 | 16.99 | 1.000 | 25.49 | 1.000 | 33.98 |
| Propanoate metabolism | 1.000 | 15.97 | 1.000 | 21.30 | 1.000 | 19.97 |
| Tyrosine metabolism | 1.000 | 12.10 | 1.000 | 14.12 | 1.000 | 15.63 |
| Starch and sucrose metabolism | 1.000 | 17.92 | 1.000 | 17.92 | 1.000 | 17.92 |
| Aminoacyl-tRNA biosynthesis | 1.000 | 13.61 | 1.000 | 20.42 | 1.000 | 21.55 |
| Ferroptosis | 1.000 | 14.59 | 1.000 | 29.17 | 1.000 | 25.53 |
| Thermogenesis | 1.000 | 17.39 | 1.000 | 17.39 | 1.000 | 17.39 |
| Glycolysis / Gluconeogenesis | 1.000 | 15.96 | 1.000 | 25.53 | 1.000 | 25.53 |
| Cysteine and methionine metabolism | 1.000 | 13.14 | 1.000 | 17.00 | 1.000 | 14.68 |
| Pathways in cancer | 1.000 | 15.96 | 1.000 | 22.34 | 1.000 | 25.53 |
| ABC transporters | 1.000 | 7.68 | 1.000 | 10.29 | 1.000 | 10.13 |
| Sphingolipid metabolism | 1.000 | 14.72 | 1.000 | 24.54 | 1.000 | 24.54 |
| AMPK signaling pathway | 1.000 | 12.67 | 1.000 | 25.35 | 1.000 | 25.35 |
| Valine, leucine and isoleucine degradation | 1.000 | 13.91 | 1.000 | 20.86 | 1.000 | 20.86 |
| Phosphonate and phosphinate metabolism | 1.000 | 8.80 | 1.000 | 18.58 | 1.000 | 17.60 |
| Thiamine metabolism | 1.000 | 12.77 | 1.000 | 19.15 | 1.000 | 19.15 |
| Pyrimidine metabolism | 1.000 | 7.99 | 1.000 | 13.79 | 1.000 | 16.70 |
| Folate biosynthesis | 1.000 | 6.61 | 1.000 | 17.94 | 1.000 | 16.05 |
| Glyoxylate and dicarboxylate metabolism | 1.000 | 11.17 | 1.000 | 15.16 | 1.000 | 15.96 |
| Terpenoid backbone biosynthesis | 1.000 | 12.12 | 1.000 | 15.15 | 1.000 | 10.60 |
| Biotin metabolism | 1.000 | 7.82 | 1.000 | 15.65 | 1.000 | 15.65 |
| Inositol phosphate metabolism | 1.000 | 9.72 | 1.000 | 11.11 | 1.000 | 13.88 |
| Glutathione metabolism | 1.000 | 8.50 | 1.000 | 10.62 | 1.000 | 8.50 |
| Glycerolipid metabolism | 1.000 | 6.37 | 1.000 | 14.87 | 1.000 | 14.87 |
| Drug metabolism - other enzymes | 1.000 | 5.67 | 1.000 | 13.61 | 1.000 | 13.61 |
| Drug metabolism - cytochrome P450 | 1.000 | 6.89 | 1.000 | 9.32 | 1.000 | 7.70 |
| Steroid hormone biosynthesis | 1.000 | 6.26 | 1.000 | 8.14 | 1.000 | 8.14 |
| Biosynthesis of unsaturated fatty acids | 1.000 | 5.04 | 1.000 | 10.64 | 1.000 | 10.64 |
| Fatty acid biosynthesis | 1.000 | 5.47 | 1.000 | 5.47 | 1.000 | 5.47 |
| Glycerophospholipid metabolism | 1.000 | 4.54 | 1.000 | 6.81 | 1.000 | 6.81 |
| Bile secretion | 1.000 | 5.54 | 1.000 | 8.48 | 1.000 | 6.85 |
| Fatty acid degradation | 1.000 | 2.45 | 1.000 | 2.45 | 1.000 | 3.68 |
| Ubiquinone and other terpenoid-quinone biosynthesis | 1.000 | 4.35 | 1.000 | 6.88 | 1.000 | 7.25 |
| Purine metabolism | 1.000 | 4.08 | 1.000 | 7.14 | 1.000 | 6.46 |
| Metabolism of xenobiotics by cytochrome P450 | 1.000 | 4.19 | 1.000 | 5.87 | 1.000 | 4.82 |
| Chemical carcinogenesis | 1.000 | 3.13 | 1.000 | 5.01 | 1.000 | 5.01 |
| Amino sugar and nucleotide sugar metabolism | 1.000 | 2.89 | 1.000 | 5.00 | 1.000 | 4.47 |
| Neomycin, kanamycin and gentamicin biosynthesis | 1.000 | 1.40 | 1.000 | 4.21 | 1.000 | 3.74 |
| Porphyrin and chlorophyll metabolism | 1.000 | 1.83 | 1.000 | 2.74 | 1.000 | 2.28 |
| **Hydrophobic metabolites involved pathway** |  |  |  |  |  |  |
| Monoradylglycerols [GL01] | 0.000 | 414.70 | 0.000 | 459.50 | 0.000 |  |
| Quinones and hydroquinones [PR02] |  |  |  |  | 0.013 |  |
| Sphingoid bases [SP01] | 0.020 | 161.30 | 0.008 | 223.70 | 0.980 |  |
| Polyether antibiotics [PK09] | 0.995 | 105.50 |  |  | 0.031 |  |
| Other Sphingolipids [SP00] |  |  |  |  | 0.208 |  |
| Phenolic lipids [PK15] | 0.739 | 150.50 | 0.558 | 243.20 | 0.553 |  |
| Polyprenols [PR03] | 0.938 | 186.30 | 0.530 | 447.20 | 0.516 |  |
| Fatty acyl glycosides [FA13] | 0.582 | 141.30 | 0.534 | 207.80 | 0.982 |  |
| Glycosyldiradylglycerols [GL05] | 0.969 | 149.20 | 0.895 | 298.40 | 0.893 |  |
| Hopanoids [PR04] | 0.997 | 95.70 | 0.998 | 191.50 | 0.952 |  |

CHD: Congenital heart disease. TOF: Tetralogy of Fallot. IAS: Inherited arrhythmias syndromes. CMP: Cardiomyopathies.

**Table S3.** Univariate analysis result of target metabolism.

| **Metabolites** | **Base Mean** | **P-value** | **Fold Change** | **AUC** |
| --- | --- | --- | --- | --- |
| Alanine | 1.30 | 0.94 | 0.99 | 0.55 |
| Asparagine | 0.66 | 0.07 | 0.95 | 0.77 |
| C0-Carnitine | 20.91 | 0.19 | 1.24 | 0.71 |
| C10-Carnitine | 1.28 | 0.01 | 0.53 | 0.81 |
| C10:1-Carnitine | 0.95 | 0.60 | 0.92 | 0.64 |
| C12-Carnitine | 3.14 | 0.00 | 0.41 | 0.92 |
| C12:1-Carnitine | 1.74 | 0.00 | 0.51 | 0.88 |
| C12:1OH-Carnitine | 0.84 | 0.02 | 0.79 | 0.81 |
| C14-Carnitine | 17.19 | 0.00 | 0.61 | 0.92 |
| C14:1-Carnitine | 6.39 | 0.00 | 0.43 | 0.91 |
| C14:1OH-Carnitine | 4.56 | 0.01 | 0.73 | 0.88 |
| C14:2-Carnitine | 1.52 | 0.02 | 0.71 | 0.77 |
| C16-Carnitine | 35.21 | 0.24 | 0.84 | 0.57 |
| C16:1-Carnitine | 11.13 | 0.08 | 0.74 | 0.75 |
| C18-Carnitine | 21.15 | 0.64 | 0.94 | 0.56 |
| C18:1-Carnitine | 11.79 | 0.60 | 0.92 | 0.55 |
| C18:2-Carnitine | 3.69 | 0.34 | 1.75 | 0.65 |
| C2-Carnitine | 80.37 | 0.41 | 0.83 | 0.53 |
| C3-Carnitine | 1.30 | 0.94 | 0.99 | 0.55 |
| C4-Carnitine | 1.30 | 0.94 | 0.99 | 0.55 |
| C5-Carnitine | 1.30 | 0.94 | 0.99 | 0.55 |
| C5DC-Carnitine/C6OH-Carnitine | 11.40 | 0.02 | 0.71 | 0.83 |
| C5OH-Carnitine | 44.04 | 0.10 | 1.27 | 0.83 |
| C6-Carnitine | 1.30 | 0.94 | 0.99 | 0.55 |
| C8-Carnitine | 1.30 | 0.94 | 0.99 | 0.55 |
| C8:1-Carnitine | 6.28 | 0.56 | 1.11 | 0.63 |
| Creatinine | 0.63 | 0.55 | 0.94 | 0.63 |
| Glutamine/Lysine | 6.69 | 0.36 | 0.85 | 0.77 |
| Hexose | 0.16 | 0.09 | 1.48 | 0.73 |
| Leucine/Isoleucine | 1.46 | 0.80 | 0.95 | 0.71 |
| Phenylalanine | 1.62 | 0.76 | 0.96 | 0.59 |
| Proline | 1.30 | 0.94 | 0.99 | 0.55 |
| Pyroglutamic acid | 1.30 | 0.94 | 0.99 | 0.55 |
| Threonine | 1.30 | 0.94 | 0.99 | 0.55 |
| Tryptophan | 0.31 | 0.38 | 1.09 | 0.68 |
| Tyrosine | 1.30 | 0.94 | 0.99 | 0.55 |
| Uric Acid | 1.30 | 0.94 | 0.99 | 0.55 |
| Valine | 3.05 | 0.75 | 0.95 | 0.73 |
| Cholesterol | 0.26 | 0.84 | 0.98 | 0.51 |
| d14:0 CE | 0.14 | 0.85 | 0.96 | 0.56 |
| d15:0 CE | 0.17 | 0.56 | 0.90 | 0.57 |
| d16:0 CE | 0.15 | 0.65 | 0.93 | 0.67 |
| d16:1 CE | 0.32 | 0.46 | 0.86 | 0.68 |
| d17:0 CE | 0.35 | 0.55 | 0.92 | 0.60 |
| d18:0 CE | 0.23 | 0.73 | 0.94 | 0.59 |
| d18:1 CE | 0.14 | 0.92 | 0.98 | 0.60 |
| d18:2 CE | 0.02 | 0.69 | 1.11 | 0.52 |
| d18:3 CE | 0.03 | 0.78 | 1.08 | 0.53 |
| d19:0 CE | 0.63 | 0.69 | 0.93 | 0.60 |
| d19:1 CE | 0.22 | 0.71 | 0.93 | 0.61 |
| d20:0 CE | 0.17 | 0.55 | 1.19 | 0.51 |
| d20:2 CE | 0.15 | 0.38 | 1.20 | 0.59 |
| d20:3 CE | 0.08 | 0.86 | 1.04 | 0.55 |
| d20:4 CE | 0.07 | 0.29 | 0.77 | 0.67 |
| d20:5 CE | 0.03 | 0.65 | 0.85 | 0.69 |
| d22:0 CE | 0.41 | 0.29 | 1.28 | 0.63 |
| d22:1 CE | 0.16 | 0.54 | 1.15 | 0.59 |
| d22:2 CE | 0.04 | 0.44 | 0.85 | 0.65 |
| d22:3 CE | 0.10 | 0.95 | 0.99 | 0.60 |
| d22:4 CE | 0.22 | 0.96 | 1.01 | 0.55 |
| d22:5 CE | 0.19 | 0.88 | 1.03 | 0.56 |
| d22:6 CE | 0.13 | 0.66 | 0.90 | 0.57 |
| d24:0 CE | 0.15 | 0.12 | 0.75 | 0.76 |
| DG(16:0-18:1) | 0.39 | 0.44 | 0.81 | 0.69 |
| DG(16:0-20:0) | 0.55 | 0.03 | 0.79 | 0.87 |
| DG(18:0-18:2) | 0.22 | 0.76 | 0.94 | 0.67 |
| DG(18:0-20:3) | 1.51 | 0.56 | 1.09 | 0.57 |
| DG(18:0-20:4) | 1.13 | 0.58 | 0.94 | 0.60 |
| DG(18:1-18:2) | 0.11 | 0.76 | 1.15 | 0.55 |
| TG(12:0-16:0-18:1) | 0.24 | 0.92 | 1.12 | 0.83 |
| TG(12:0-16:0-18:2) | 0.31 | 0.12 | 0.55 | 0.84 |
| TG(12:0-18:0-18:2) | 0.25 | 0.12 | 0.53 | 0.83 |
| TG(12:0-18:1-18:1)/TG(14:0-16:1-18:1) | 0.23 | 0.99 | 0.99 | 0.72 |
| TG(12:0-18:1-18:2) | 0.09 | 0.62 | 3.20 | 0.63 |
| TG(12:0-18:2-18:2) | 0.07 | 0.91 | 0.92 | 0.76 |
| TG(14:0-14:0-16:0) | 0.37 | 0.26 | 0.74 | 0.80 |
| TG(14:0-14:0-16:1) | 0.41 | 0.98 | 0.99 | 0.81 |
| TG(14:0-14:0-18:0) | 0.41 | 0.89 | 0.92 | 0.77 |
| TG(14:0-14:0-18:1)/TG(14:0-16:0-16:1) | 0.36 | 0.08 | 0.68 | 0.76 |
| TG(14:0-16:0-16:0) | 0.46 | 0.06 | 0.66 | 0.79 |
| TG(14:0-16:0-18:1)/TG(16:0-16:0-16:1) | 0.73 | 0.13 | 0.68 | 0.72 |
| TG(14:0-16:0-18:2)/TG(16:0-16:1-16:1) | 0.35 | 0.19 | 0.71 | 0.69 |
| TG(14:0-16:0-18:3) | 0.06 | 0.97 | 1.02 | 0.64 |
| TG(14:0-16:0-20:4) | 0.18 | 0.16 | 0.66 | 0.81 |
| TG(14:0-16:1-18:0) | 0.32 | 0.64 | 0.83 | 0.72 |
| TG(14:0-16:1-18:2) | 0.53 | 0.13 | 0.70 | 0.76 |
| TG(14:0-18:0-18:2) | 0.31 | 0.29 | 0.77 | 0.69 |
| TG(14:0-18:1-18:1)/TG(16:0-16:1-18:1) | 0.42 | 0.78 | 0.92 | 0.63 |
| TG(14:0-18:1-18:2) | 0.05 | 0.41 | 1.52 | 0.57 |
| TG(14:0-18:1-18:3) | 0.07 | 0.66 | 0.88 | 0.69 |
| TG(14:0-18:2-18:2)/TG(16:1-16:1-18:2) | 0.06 | 0.68 | 1.13 | 0.53 |
| TG(14:0-18:2-18:3) | 0.06 | 0.65 | 0.89 | 0.63 |
| TG(16:0-16:0-18:1) | 0.51 | 0.45 | 0.83 | 0.61 |
| TG(16:0-16:0-18:2) | 0.34 | 0.78 | 0.92 | 0.60 |
| TG(16:0-16:0-18:3) | 0.12 | 0.86 | 1.04 | 0.52 |
| TG(16:0-16:0-20:1) | 0.32 | 0.71 | 0.92 | 0.59 |
| TG(16:0-16:0-20:2) | 0.54 | 0.58 | 0.87 | 0.61 |
| TG(16:0-16:0-20:3) | 0.71 | 0.82 | 0.92 | 0.63 |
| TG(16:0-16:0-20:4) | 0.44 | 0.30 | 0.70 | 0.73 |
| TG(16:0-16:0-20:5) | 0.31 | 0.09 | 0.57 | 0.72 |
| TG(16:0-16:0-22:4) | 0.72 | 0.78 | 0.90 | 0.71 |
| TG(16:0-16:0-22:5) | 1.09 | 0.88 | 0.93 | 0.68 |
| TG(16:0-16:0-22:6) | 2.06 | 0.08 | 0.60 | 0.77 |
| TG(16:0-16:1-18:2)/TG(16:1-16:1-18:1) | 0.29 | 0.67 | 0.88 | 0.64 |
| TG(16:0-16:1-18:3) | 0.12 | 0.57 | 0.87 | 0.75 |
| TG(16:0-16:1-20:1) | 0.66 | 0.77 | 0.92 | 0.53 |
| TG(16:0-16:1-20:2) | 0.33 | 0.89 | 1.04 | 0.53 |
| TG(16:0-16:1-20:3) | 0.68 | 0.90 | 1.05 | 0.52 |
| TG(16:0-16:1-20:4) | 0.52 | 0.56 | 0.80 | 0.68 |
| TG(16:0-16:1-22:4) | 0.72 | 0.98 | 0.99 | 0.60 |
| TG(16:0-16:1-22:5) | 1.26 | 0.82 | 1.12 | 0.60 |
| TG(16:0-16:2-20:1)/TG(16:1-16:1-20:1) | 0.20 | 0.63 | 0.90 | 0.61 |
| TG(16:0-16:2-20:2)/TG(16:1-16:1-20:2) | 0.21 | 0.82 | 0.94 | 0.60 |
| TG(16:0-16:2-20:3)/TG(16:1-16:1-20:3) | 0.39 | 0.57 | 0.83 | 0.71 |
| TG(16:0-18:1-18:1) | 0.23 | 0.88 | 1.04 | 0.52 |
| TG(16:0-18:1-18:2)/TG(16:1-18:0-18:2) | 0.07 | 0.31 | 1.37 | 0.67 |
| TG(16:0-18:1-18:3) | 0.06 | 0.42 | 1.25 | 0.53 |
| TG(16:0-18:1-20:2) | 0.26 | 0.76 | 1.09 | 0.51 |
| TG(16:0-18:1-20:3)/TG(16:1-18:0-20:3) | 0.33 | 0.79 | 1.11 | 0.55 |
| TG(16:0-18:1-20:4) | 0.22 | 0.66 | 0.85 | 0.71 |
| TG(16:0-18:1-20:5) | 0.16 | 0.03 | 0.56 | 0.79 |
| TG(16:0-18:1-22:5) | 0.50 | 0.77 | 1.15 | 0.64 |
| TG(16:0-18:1-22:6) | 0.98 | 0.37 | 0.75 | 0.67 |
| TG(16:0-18:2-18:2)/TG(16:1-18:1-18:2) | 0.04 | 0.20 | 1.60 | 0.65 |
| TG(16:0-18:2-18:3)/TG(16:1-18:2-18:2) | 0.03 | 0.25 | 1.58 | 0.64 |
| TG(16:0-18:2-20:2)/TG(16:1-18:1-20:2) | 0.15 | 0.56 | 1.17 | 0.56 |
| TG(16:0-18:2-20:3)/TG(16:1-18:1-20:3) | 0.21 | 0.54 | 1.27 | 0.55 |
| TG(16:0-18:2-20:4)/TG(16:1-18:1-20:4) | 0.14 | 0.93 | 0.97 | 0.68 |
| TG(16:0-18:2-22:5)/TG(16:1-18:1-22:5) | 0.31 | 0.64 | 1.24 | 0.56 |
| TG(16:0-18:3-20:2)/TG(16:1-18:2-20:2) | 0.10 | 0.59 | 1.20 | 0.56 |
| TG(16:0-18:3-20:3)/TG(16:1-18:2-20:3) | 0.16 | 0.62 | 1.22 | 0.56 |
| TG(16:0-18:3-20:3)/TG(18:1-18:2-18:3) | 0.05 | 0.40 | 1.35 | 0.59 |
| TG(16:0-18:3-22:4)/TG(16:1-18:2-22:4) | 0.15 | 0.47 | 1.55 | 0.53 |
| TG(16:0-20:2-20:4)/TG(18:0-18:2-20:4)/TG(18:1-18:1-20:4) | 0.14 | 0.93 | 0.97 | 0.67 |
| TG(16:0-20:3-20:4) | 0.09 | 0.48 | 1.27 | 0.56 |
| TG(16:0-20:4-22:4) | 0.51 | 0.28 | 0.81 | 0.71 |
| TG(16:1-16:1-20:4) | 0.28 | 0.13 | 0.62 | 0.73 |
| TG(16:1-18:1-18:1)/TG(16:2-18:0-18:1) | 0.11 | 0.70 | 1.12 | 0.56 |
| TG(16:1-18:2-18:3) | 0.04 | 0.77 | 1.06 | 0.52 |
| TG(16:1-18:2-20:1) | 0.13 | 0.33 | 1.51 | 0.64 |
| TG(16:1-20:3-20:3)/TG(16:1-18:1-22:5)/TG(16:1-18:2-22:4) | 0.54 | 0.89 | 0.95 | 0.61 |
| TG(16:2-16:1-20:0) | 0.07 | 0.35 | 1.30 | 0.64 |
| TG(16:2-16:1-20:1) | 0.06 | 0.50 | 1.42 | 0.56 |
| TG(16:2-18:1-18:2)/TG(16:1-18:1-18:3) | 0.06 | 0.99 | 1.00 | 0.53 |
| TG(17:0-18:1-18:1) | 0.23 | 0.80 | 1.06 | 0.53 |
| TG(17:0-18:1-18:2) | 0.05 | 0.45 | 1.23 | 0.60 |
| TG(18:0-18:2-18:2)/TG(18:1-18:1-18:2)/TG(16:0-18:2-20:2) | 0.07 | 0.37 | 1.52 | 0.53 |
| TG(18:0-18:2-20:2) | 0.24 | 0.60 | 0.88 | 0.63 |
| TG(18:1-18:1-18:3)/TG(16:0-18:3-20:2) | 0.06 | 0.52 | 1.33 | 0.55 |
| TG(18:1-18:1-20:0) | 0.41 | 0.59 | 1.22 | 0.53 |
| TG(18:1-18:1-20:1) | 0.43 | 0.54 | 1.31 | 0.56 |
| TG(18:1-18:1-20:3) | 0.21 | 0.78 | 1.10 | 0.56 |
| TG(18:1-18:2-20:3)/TG(18:0-18:3-20:3) | 0.13 | 0.26 | 1.61 | 0.61 |
| TG(18:1-18:3-20:2)/TG(18:2-18:2-20:2)/TG(16:0-20:2-20:4) | 0.09 | 0.54 | 1.36 | 0.51 |
| TG(18:1-18:3-20:3)/TG(16:0-18:3-22:4) | 0.07 | 0.46 | 1.37 | 0.65 |
| TG(18:2-18:2-18:3) | 0.09 | 0.82 | 0.94 | 0.53 |
| TG(18:2-18:2-20:3)/TG(16:0-18:2-22:5)/TG(16:1-18:2-22:4) | 0.07 | 0.25 | 1.55 | 0.63 |
| Cer(d18:1-12:0) | 0.15 | 0.03 | 0.83 | 0.85 |
| Cer(d18:1-13:0) | 0.11 | 0.68 | 0.94 | 0.53 |
| Cer(d18:1-16:0) | 3.67 | 0.55 | 0.93 | 0.60 |
| Cer(d18:1-18:0) | 7.89 | 0.95 | 0.99 | 0.57 |
| Cer(d18:1-20:0) | 4.23 | 0.74 | 1.05 | 0.59 |
| Cer(d18:1-22:0) | 2.75 | 0.44 | 0.93 | 0.64 |
| Cer(d18:1-23:0) | 0.40 | 0.52 | 0.92 | 0.60 |
| Cer(d18:1-24:0) | 2.28 | 0.32 | 0.90 | 0.64 |
| Cer(d18:1-24:1) | 6.89 | 0.55 | 1.10 | 0.57 |
| Cer(d18:1-24:2) | 13.35 | 0.71 | 0.95 | 0.56 |
| Cer(d18:1-25:0) | 1.14 | 1.00 | 1.00 | 0.57 |
| Cer(OH-d18:1-24:0) | 1.95 | 0.93 | 0.99 | 0.52 |
| CerP(d18:1-12:0) | 0.35 | 0.30 | 1.09 | 0.63 |
| CerP(d18:1-14:0) | 0.36 | 0.18 | 1.11 | 0.72 |
| CerP(d18:1-16:0) | 0.50 | 0.17 | 1.10 | 0.72 |
| CerP(d18:1-16:1) | 0.35 | 0.56 | 1.04 | 0.63 |
| CerP(d18:1-18:0) | 0.58 | 0.90 | 0.99 | 0.52 |
| CerP(d18:1-18:1) | 0.53 | 0.40 | 1.05 | 0.60 |
| CerP(d18:1-20:0) | 0.31 | 0.09 | 1.10 | 0.73 |
| CerP(d18:1-22:0) | 0.35 | 0.83 | 0.99 | 0.52 |
| LysoPA(16:0) | 0.07 | 0.71 | 1.07 | 0.63 |
| LysoPA(18:0) | 0.07 | 0.64 | 0.91 | 0.63 |
| LysoPA(18:1) | 0.14 | 0.85 | 1.09 | 0.68 |
| LysoPA(18:2) | 0.08 | 0.62 | 1.28 | 0.57 |
| LysoPE(d16:1) | 1.51 | 0.06 | 1.34 | 0.79 |
| LysoPE(d18:0) | 0.30 | 0.42 | 1.10 | 0.65 |
| LysoPE(d18:1) | 0.97 | 0.06 | 1.39 | 0.79 |
| LysoPE(d18:2) | 0.25 | 0.07 | 1.64 | 0.89 |
| LysoPE(d20:1) | 2.05 | 0.09 | 1.55 | 0.76 |
| LysoPE(d20:3) | 2.10 | 0.03 | 1.68 | 0.87 |
| LysoPE(d20:4) | 2.01 | 0.26 | 1.21 | 0.71 |
| LysoPG(d16:0) | 0.30 | 0.54 | 0.93 | 0.60 |
| LysoPG(d18:0) | 0.84 | 0.71 | 1.03 | 0.51 |
| LysoPG(d18:1) | 1.01 | 0.12 | 0.83 | 0.75 |
| LysoPG(d18:2) | 0.45 | 0.07 | 0.69 | 0.76 |
| PA(d14:0-14:0) | 3.28 | 0.91 | 1.04 | 0.53 |
| PA(d14:1-14:1) | 0.57 | 0.49 | 1.05 | 0.64 |
| PA(d16:0-18:1) | 1.70 | 0.70 | 1.04 | 0.63 |
| PA(d16:0-18:2) | 0.39 | 0.35 | 1.14 | 0.67 |
| PA(d16:0-20:3) | 0.24 | 0.13 | 1.28 | 0.75 |
| PA(d16:0-20:4) | 1.00 | 0.13 | 0.79 | 0.73 |
| PA(d16:1-18:1) | 2.27 | 0.84 | 1.03 | 0.63 |
| PA(d18:0-18:1) | 1.59 | 0.84 | 1.03 | 0.52 |
| PA(d18:0-20:2) | 0.38 | 0.48 | 1.08 | 0.63 |
| PA(d18:0-20:3) | 0.36 | 0.18 | 1.23 | 0.63 |
| PA(d18:1-18:1) | 0.73 | 0.43 | 1.09 | 0.64 |
| PA(d18:1-18:3) | 0.79 | 0.65 | 0.95 | 0.56 |
| PA(d18:1-20:2) | 0.40 | 0.32 | 1.17 | 0.73 |
| PA(d18:1-20:3) | 0.21 | 0.17 | 1.34 | 0.72 |
| PA(d18:2-18:0) | 0.19 | 0.92 | 1.02 | 0.51 |
| PA(d18:2-18:1) | 0.15 | 0.08 | 1.40 | 0.81 |
| PA(d18:2-18:2) | 0.11 | 0.42 | 1.19 | 0.69 |
| PA(d18:2-20:3) | 0.04 | 0.34 | 1.40 | 0.61 |
| PA(d20:2-20:2) | 0.30 | 0.77 | 1.06 | 0.52 |
| PE(a14:0-20:2) | 0.90 | 0.52 | 1.16 | 0.63 |
| PE(a14:1-24:5) | 0.99 | 0.47 | 1.03 | 0.61 |
| PE(a16:0-18:1) | 2.77 | 0.47 | 1.07 | 0.65 |
| PE(a16:0-20:1) | 1.28 | 0.54 | 1.07 | 0.65 |
| PE(a16:0-20:2) | 1.34 | 0.64 | 1.05 | 0.61 |
| PE(a16:0-20:3) | 0.30 | 0.07 | 1.32 | 0.81 |
| PE(a16:0-20:4) | 1.01 | 0.66 | 0.93 | 0.61 |
| PE(a16:0-20:5) | 0.51 | 0.39 | 0.91 | 0.68 |
| PE(a16:0-22:1) | 1.23 | 0.47 | 1.08 | 0.65 |
| PE(a16:0-22:2) | 0.31 | 0.05 | 1.37 | 0.85 |
| PE(a16:0-22:3) | 1.23 | 0.93 | 1.01 | 0.51 |
| PE(a16:0-22:4) | 0.44 | 0.56 | 1.07 | 0.56 |
| PE(a16:0-22:5) | 0.11 | 0.12 | 0.83 | 0.76 |
| PE(a16:0-22:6) | 0.64 | 0.85 | 0.96 | 0.59 |
| PE(a16:0-24:0) | 0.52 | 0.16 | 1.22 | 0.75 |
| PE(a16:0-26:2) | 0.78 | 0.57 | 0.95 | 0.57 |
| PE(a16:1-18:0) | 3.23 | 0.36 | 1.15 | 0.68 |
| PE(a16:1-18:2) | 4.32 | 0.55 | 1.10 | 0.60 |
| PE(a16:1-20:0) | 4.14 | 0.96 | 0.99 | 0.53 |
| PE(a16:1-20:2) | 1.94 | 0.65 | 1.07 | 0.64 |
| PE(a16:1-20:3) | 0.60 | 0.87 | 1.03 | 0.51 |
| PE(a16:1-20:4) | 0.80 | 0.91 | 1.02 | 0.53 |
| PE(a16:1-22:1) | 1.37 | 0.72 | 1.07 | 0.51 |
| PE(a16:1-22:2) | 0.52 | 0.52 | 1.11 | 0.63 |
| PE(a18:0-18:1) | 1.83 | 0.77 | 1.04 | 0.57 |
| PE(a18:0-18:2) | 0.26 | 0.28 | 1.13 | 0.68 |
| PE(a18:0-20:0) | 2.34 | 0.27 | 1.13 | 0.71 |
| PE(a18:0-20:1) | 0.55 | 0.29 | 1.12 | 0.68 |
| PE(a18:0-20:2) | 0.96 | 0.87 | 0.99 | 0.51 |
| PE(a18:0-20:3) | 0.22 | 0.22 | 1.15 | 0.68 |
| PE(a18:0-20:4) | 0.78 | 0.67 | 0.94 | 0.59 |
| PE(a18:0-20:5) | 0.50 | 0.91 | 0.98 | 0.51 |
| PE(a18:0-22:0) | 8.66 | 0.52 | 1.12 | 0.61 |
| PE(a18:0-22:1) | 1.42 | 0.87 | 0.98 | 0.56 |
| PE(a18:0-22:2) | 0.20 | 0.30 | 1.16 | 0.65 |
| PE(a18:0-22:3) | 0.72 | 0.64 | 0.95 | 0.59 |
| PE(a18:0-22:4) | 0.46 | 0.77 | 1.03 | 0.53 |
| PE(a18:0-22:5) | 0.12 | 0.05 | 0.81 | 0.77 |
| PE(a18:0-22:6) | 0.58 | 0.50 | 0.92 | 0.61 |
| PE(a18:1-18:1) | 1.58 | 0.58 | 1.07 | 0.55 |
| PE(a18:1-18:2) | 1.60 | 0.50 | 1.15 | 0.60 |
| PE(a18:1-18:3) | 0.43 | 0.17 | 1.25 | 0.73 |
| PE(a18:1-20:0) | 1.28 | 0.59 | 1.06 | 0.67 |
| PE(a18:1-20:1) | 1.28 | 0.87 | 1.02 | 0.52 |
| PE(a18:1-20:2) | 1.20 | 0.43 | 1.13 | 0.61 |
| PE(a18:1-20:3) | 0.38 | 0.15 | 1.26 | 0.77 |
| PE(a18:1-20:4) | 0.85 | 0.78 | 0.95 | 0.59 |
| PE(a18:1-20:5) | 1.18 | 0.99 | 1.00 | 0.53 |
| PE(a18:1-22:0) | 1.14 | 0.74 | 1.04 | 0.52 |
| PE(a18:1-22:1) | 0.92 | 0.39 | 1.13 | 0.65 |
| PE(a18:1-22:2) | 0.38 | 0.18 | 1.26 | 0.68 |
| PE(a18:1-22:3) | 1.57 | 0.53 | 1.11 | 0.55 |
| PE(a18:1-22:4) | 0.69 | 0.59 | 1.07 | 0.53 |
| PE(a18:1-22:5) | 0.15 | 0.19 | 0.85 | 0.75 |
| PE(a18:1-24:1) | 3.16 | 0.34 | 1.19 | 0.61 |
| PE(a18:2-18:2) | 0.17 | 0.22 | 1.24 | 0.72 |
| PE(a18:2-20:0) | 0.26 | 0.05 | 1.42 | 0.87 |
| PE(a18:2-20:1) | 0.16 | 0.21 | 1.19 | 0.69 |
| PE(a18:2-20:2) | 0.15 | 0.14 | 1.40 | 0.80 |
| PE(a18:2-20:3) | 0.08 | 0.37 | 1.17 | 0.64 |
| PE(a18:2-22:0) | 0.16 | 0.15 | 1.26 | 0.75 |
| PE(a18:2-22:1) | 0.15 | 0.11 | 1.45 | 0.80 |
| PE(a18:2-22:2) | 0.09 | 0.15 | 1.35 | 0.75 |
| PE(a18:2-22:3) | 0.26 | 0.39 | 1.15 | 0.64 |
| PE(a18:2-22:4) | 0.15 | 0.60 | 1.07 | 0.59 |
| PE(a18:2-24:0) | 0.27 | 0.55 | 1.10 | 0.59 |
| PE(a18:3-20:0) | 0.43 | 0.15 | 1.41 | 0.75 |
| PE(a18:3-20:1) | 0.30 | 0.74 | 1.13 | 0.53 |
| PE(a20:0-20:1) | 1.24 | 0.57 | 1.07 | 0.61 |
| PE(a20:0-20:2) | 0.58 | 0.02 | 1.37 | 0.89 |
| PE(a20:0-20:3) | 0.52 | 0.14 | 1.25 | 0.72 |
| PE(a20:0-20:4) | 0.67 | 0.26 | 0.88 | 0.65 |
| PE(a20:0-20:5) | 0.28 | 0.38 | 0.83 | 0.69 |
| PE(a20:0-22:5) | 0.44 | 0.31 | 1.16 | 0.63 |
| PE(a20:1-20:2) | 0.69 | 0.07 | 1.36 | 0.77 |
| PE(a20:1-20:3) | 0.56 | 0.34 | 1.13 | 0.63 |
| PE(a20:1-20:4) | 1.05 | 0.45 | 0.91 | 0.67 |
| PE(a20:1-20:5) | 0.34 | 0.80 | 1.07 | 0.52 |
| PE(a20:2-20:3) | 0.72 | 0.22 | 1.31 | 0.72 |
| PE(a20:2-22:0) | 0.38 | 0.26 | 1.25 | 0.67 |
| PE(a20:2-22:6) | 1.55 | 0.75 | 0.93 | 0.55 |
| PE(a20:4-22:4) | 0.68 | 0.90 | 0.98 | 0.65 |
| PE(d14:0-20:0) | 1.07 | 0.63 | 1.09 | 0.61 |
| PE(d14:0-20:1) | 1.00 | 0.93 | 0.99 | 0.52 |
| PE(d14:0-20:2) | 0.28 | 0.11 | 2.13 | 0.75 |
| PE(d14:0-22:3) | 3.76 | 0.51 | 0.93 | 0.60 |
| PE(d16:0-18:0) | 3.76 | 0.62 | 1.04 | 0.63 |
| PE(d16:0-18:1) | 6.88 | 0.73 | 1.05 | 0.56 |
| PE(d16:0-18:2) | 1.10 | 0.49 | 1.10 | 0.64 |
| PE(d16:0-20:0) | 1.35 | 0.84 | 0.98 | 0.51 |
| PE(d16:0-20:1) | 1.54 | 0.82 | 1.02 | 0.57 |
| PE(d16:0-20:2) | 0.31 | 0.06 | 1.31 | 0.83 |
| PE(d16:0-20:3) | 2.98 | 0.28 | 1.23 | 0.68 |
| PE(d16:0-20:4) | 3.93 | 0.76 | 0.95 | 0.53 |
| PE(d16:0-22:0) | 0.17 | 0.94 | 0.99 | 0.52 |
| PE(d16:0-22:2) | 2.83 | 0.44 | 1.08 | 0.63 |
| PE(d16:0-22:3) | 1.72 | 0.21 | 1.13 | 0.76 |
| PE(d16:0-22:4) | 1.26 | 0.74 | 0.96 | 0.56 |
| PE(d16:0-22:5) | 1.19 | 0.74 | 1.04 | 0.61 |
| PE(d16:0-22:6) | 2.12 | 0.86 | 0.97 | 0.52 |
| PE(d16:1-18:0) | 6.88 | 0.73 | 1.05 | 0.56 |
| PE(d16:1-18:1) | 3.19 | 0.22 | 1.35 | 0.68 |
| PE(d16:1-18:2) | 0.28 | 0.80 | 1.03 | 0.56 |
| PE(d16:1-20:0) | 2.53 | 0.59 | 1.07 | 0.68 |
| PE(d16:1-20:1) | 1.93 | 0.86 | 1.03 | 0.59 |
| PE(d16:1-20:2) | 0.43 | 0.08 | 1.32 | 0.76 |
| PE(d16:1-20:4) | 0.66 | 0.77 | 1.05 | 0.56 |
| PE(d16:1-22:2) | 4.33 | 0.87 | 1.02 | 0.52 |
| PE(d16:1-22:3) | 4.14 | 0.84 | 0.97 | 0.52 |
| PE(d16:1-22:4) | 2.24 | 0.30 | 1.20 | 0.65 |
| PE(d16:1-22:5) | 0.57 | 0.74 | 1.08 | 0.51 |
| PE(d16:1-22:6) | 0.69 | 0.48 | 0.83 | 0.61 |
| PE(d18:0-18:0) | 1.27 | 0.87 | 1.02 | 0.51 |
| PE(d18:0-18:1) | 1.71 | 0.68 | 1.05 | 0.64 |
| PE(d18:0-18:3) | 0.30 | 0.32 | 1.25 | 0.63 |
| PE(d18:0-20:0) | 1.00 | 0.62 | 0.92 | 0.59 |
| PE(d18:0-20:1) | 0.97 | 0.63 | 0.95 | 0.55 |
| PE(d18:0-20:2) | 0.19 | 0.23 | 1.16 | 0.67 |
| PE(d18:0-20:3) | 1.14 | 0.35 | 1.18 | 0.64 |
| PE(d18:0-20:4) | 1.13 | 0.68 | 0.93 | 0.59 |
| PE(d18:0-20:5) | 0.74 | 0.41 | 0.90 | 0.65 |
| PE(d18:0-22:2) | 1.42 | 0.38 | 1.10 | 0.61 |
| PE(d18:0-22:3) | 0.76 | 0.63 | 1.05 | 0.53 |
| PE(d18:0-22:4) | 0.78 | 0.55 | 0.94 | 0.60 |
| PE(d18:0-22:5) | 0.72 | 0.51 | 0.94 | 0.59 |
| PE(d18:0-22:6) | 1.00 | 0.69 | 0.94 | 0.56 |
| PE(d18:1-18:1) | 2.32 | 0.23 | 1.29 | 0.69 |
| PE(d18:1-18:3) | 1.40 | 0.94 | 0.99 | 0.55 |
| PE(d18:1-20:0) | 1.21 | 0.81 | 1.02 | 0.55 |
| PE(d18:1-20:1) | 1.34 | 0.45 | 1.13 | 0.61 |
| PE(d18:1-20:2) | 0.51 | 0.16 | 1.26 | 0.71 |
| PE(d18:1-20:3) | 3.28 | 0.25 | 1.29 | 0.68 |
| PE(d18:1-20:4) | 4.06 | 0.33 | 1.29 | 0.64 |
| PE(d18:1-22:0) | 0.32 | 0.55 | 0.93 | 0.60 |
| PE(d18:1-22:1) | 1.32 | 0.62 | 1.06 | 0.57 |
| PE(d18:1-22:2) | 1.38 | 0.36 | 1.11 | 0.67 |
| PE(d18:1-22:3) | 1.29 | 0.55 | 1.06 | 0.60 |
| PE(d18:1-22:4) | 1.68 | 0.44 | 1.13 | 0.69 |
| PE(d18:1-22:5) | 2.02 | 0.31 | 1.15 | 0.65 |
| PE(d18:1-22:6) | 5.08 | 0.56 | 1.12 | 0.52 |
| PE(d18:2-18:0) | 0.24 | 0.10 | 1.29 | 0.80 |
| PE(d18:2-18:1) | 0.32 | 0.20 | 1.29 | 0.71 |
| PE(d18:2-18:2) | 0.22 | 0.55 | 1.09 | 0.63 |
| PE(d18:2-20:0) | 0.15 | 0.37 | 1.15 | 0.61 |
| PE(d18:2-20:1) | 0.17 | 0.33 | 1.21 | 0.71 |
| PE(d18:2-20:2) | 0.07 | 0.14 | 1.45 | 0.75 |
| PE(d18:2-20:3) | 0.67 | 0.19 | 1.31 | 0.72 |
| PE(d18:2-20:4) | 1.33 | 0.87 | 1.03 | 0.56 |
| PE(d18:2-22:0) | 0.11 | 0.81 | 0.93 | 0.72 |
| PE(d18:2-22:1) | 0.24 | 0.08 | 1.34 | 0.75 |
| PE(d18:2-22:2) | 0.29 | 0.04 | 1.41 | 0.80 |
| PE(d18:2-22:3) | 0.27 | 0.09 | 1.31 | 0.75 |
| PE(d18:2-22:4) | 0.38 | 0.11 | 1.31 | 0.81 |
| PE(d18:2-22:5) | 0.45 | 0.32 | 1.18 | 0.68 |
| PE(d18:2-22:6) | 0.62 | 0.85 | 1.03 | 0.56 |
| PE(d18:3-20:0) | 0.17 | 0.49 | 1.15 | 0.59 |
| PE(d18:3-20:1) | 0.23 | 0.35 | 1.53 | 0.60 |
| PE(d18:3-20:2) | 0.25 | 0.07 | 2.45 | 0.80 |
| PE(d18:3-22:2) | 0.41 | 0.40 | 1.37 | 0.61 |
| PE(d18:3-22:3) | 0.29 | 0.81 | 1.07 | 0.52 |
| PE(d20:0-20:1) | 0.38 | 0.55 | 1.10 | 0.57 |
| PE(d20:0-20:2) | 0.92 | 0.33 | 1.17 | 0.64 |
| PE(d20:0-20:3) | 0.37 | 0.28 | 1.13 | 0.60 |
| PE(d20:0-20:4) | 0.63 | 0.05 | 0.81 | 0.76 |
| PE(d20:0-20:5) | 0.30 | 0.71 | 0.93 | 0.57 |
| PE(d20:1-20:2) | 1.10 | 0.03 | 1.72 | 0.87 |
| PE(d20:1-20:3) | 0.73 | 0.48 | 1.14 | 0.57 |
| PE(d20:1-20:4) | 1.03 | 0.86 | 0.97 | 0.56 |
| PE(d20:2-20:2) | 0.34 | 0.73 | 1.11 | 0.52 |
| PE(d20:2-20:3) | 0.55 | 0.28 | 1.28 | 0.65 |
| PE(d20:2-20:4) | 0.70 | 0.84 | 0.97 | 0.59 |
| PE(d20:3-20:3) | 3.34 | 0.10 | 1.72 | 0.76 |
| PE(p16:0-20:4) | 0.51 | 0.39 | 0.91 | 0.68 |
| PE(p16:0-20:5) | 0.26 | 0.75 | 0.90 | 0.55 |
| PE(p16:0-22:0) | 1.23 | 0.47 | 1.08 | 0.65 |
| PE(p16:0-22:1) | 0.31 | 0.05 | 1.37 | 0.85 |
| PE(p16:0-22:4) | 4.70 | 0.41 | 1.19 | 0.63 |
| PE(p16:0-22:5) | 0.55 | 0.53 | 1.13 | 0.53 |
| PE(p16:0-22:6) | 0.72 | 0.82 | 1.05 | 0.51 |
| PE(p16:0-24:1) | 1.40 | 0.40 | 1.11 | 0.65 |
| PE(p16:0-24:2) | 0.87 | 0.35 | 1.11 | 0.64 |
| PE(p16:0-24:3) | 0.64 | 0.58 | 0.94 | 0.65 |
| PE(p18:0-18:0) | 1.88 | 0.97 | 1.00 | 0.51 |
| PE(p18:0-18:1) | 1.58 | 0.58 | 1.07 | 0.55 |
| PE(p18:0-18:2) | 0.21 | 0.08 | 1.28 | 0.80 |
| PE(p18:0-18:3) | 0.37 | 0.50 | 1.15 | 0.59 |
| PE(p18:0-20:0) | 0.55 | 0.29 | 1.12 | 0.68 |
| PE(p18:0-20:1) | 0.96 | 0.87 | 0.99 | 0.51 |
| PE(p18:0-20:2) | 0.22 | 0.22 | 1.15 | 0.68 |
| PE(p18:0-20:4) | 0.85 | 0.78 | 0.95 | 0.59 |
| PE(p18:0-20:5) | 0.21 | 0.66 | 0.88 | 0.61 |
| PE(p18:0-22:0) | 1.42 | 0.87 | 0.98 | 0.56 |
| PE(p18:0-22:1) | 0.20 | 0.30 | 1.16 | 0.65 |
| PE(p18:0-22:2) | 0.72 | 0.64 | 0.95 | 0.59 |
| PE(p18:0-22:3) | 0.46 | 0.77 | 1.03 | 0.53 |
| PE(p18:0-22:4) | 0.12 | 0.05 | 0.81 | 0.77 |
| PE(p18:0-22:5) | 0.58 | 0.50 | 0.92 | 0.61 |
| PE(p18:0-22:6) | 1.00 | 0.62 | 0.92 | 0.59 |
| PE(p18:1-18:1) | 1.60 | 0.50 | 1.15 | 0.60 |
| PE(p18:1-18:2) | 0.43 | 0.17 | 1.25 | 0.73 |
| PE(p18:1-20:0) | 1.28 | 0.87 | 1.02 | 0.52 |
| PE(p18:1-20:1) | 1.20 | 0.43 | 1.13 | 0.61 |
| PE(p18:1-20:2) | 0.38 | 0.15 | 1.26 | 0.77 |
| PE(p18:1-20:3) | 0.70 | 0.14 | 1.42 | 0.73 |
| PE(p18:1-20:4) | 1.18 | 0.99 | 1.00 | 0.53 |
| PE(p18:1-20:5) | 0.16 | 0.36 | 1.33 | 0.65 |
| PE(p18:1-22:0) | 0.92 | 0.39 | 1.13 | 0.65 |
| PE(p18:1-22:1) | 0.38 | 0.18 | 1.26 | 0.68 |
| PE(p18:1-22:2) | 1.57 | 0.53 | 1.11 | 0.55 |
| PE(p18:1-22:3) | 0.69 | 0.59 | 1.07 | 0.53 |
| PE(p18:1-22:4) | 0.15 | 0.19 | 0.85 | 0.75 |
| PE(p18:1-22:5) | 1.31 | 0.94 | 1.01 | 0.56 |
| PE(p18:1-22:6) | 1.21 | 0.81 | 1.02 | 0.55 |
| PE(p18:1-24:0) | 3.16 | 0.34 | 1.19 | 0.61 |
| PE(p18:2-20:0) | 0.16 | 0.21 | 1.19 | 0.69 |
| PE(p18:2-20:1) | 0.15 | 0.14 | 1.40 | 0.80 |
| PE(p18:2-20:2) | 0.08 | 0.37 | 1.17 | 0.64 |
| PE(p18:2-22:0) | 0.15 | 0.11 | 1.45 | 0.80 |
| PE(p18:2-22:1) | 0.09 | 0.15 | 1.35 | 0.75 |
| PE(p18:2-22:2) | 0.26 | 0.39 | 1.15 | 0.64 |
| PE(p18:2-22:3) | 0.15 | 0.60 | 1.07 | 0.59 |
| PE(p18:2-22:4) | 0.07 | 0.18 | 0.82 | 0.68 |
| PE(p20:0-20:2) | 0.69 | 0.07 | 1.36 | 0.77 |
| PE(p20:0-20:3) | 0.56 | 0.34 | 1.13 | 0.63 |
| PE(p20:0-20:4) | 1.05 | 0.45 | 0.91 | 0.67 |
| PE(p20:0-20:5) | 0.34 | 0.80 | 1.07 | 0.52 |
| PE(p20:0-22:4) | 5.71 | 0.75 | 0.96 | 0.53 |
| PE(p20:1-20:3) | 0.72 | 0.22 | 1.31 | 0.72 |
| PE(p20:1-22:6) | 1.55 | 0.75 | 0.93 | 0.55 |
| PE(p20:4-22:3) | 0.68 | 0.90 | 0.98 | 0.65 |
| PG(d14:0-16:0) | 0.91 | 0.22 | 0.94 | 0.71 |
| PG(d16:0-16:0) | 0.56 | 0.10 | 0.84 | 0.79 |
| PG(d16:0-18:0) | 0.70 | 0.69 | 1.04 | 0.57 |
| PG(d16:0-18:1) | 1.20 | 0.58 | 0.95 | 0.60 |
| PG(d16:0-18:2) | 1.05 | 0.98 | 1.00 | 0.52 |
| PG(d18:0-18:1) | 0.91 | 0.75 | 1.03 | 0.51 |
| PI(d16:1-16:0) | 1.95 | 0.75 | 0.96 | 0.52 |
| PI(d18:0-18:1) | 0.58 | 0.49 | 1.06 | 0.64 |
| PI(d18:0-20:3) | 0.74 | 0.31 | 1.13 | 0.64 |
| PI(d18:0-20:4) | 0.51 | 0.53 | 0.94 | 0.60 |
| PI(d18:0-22:4) | 0.88 | 0.45 | 1.09 | 0.63 |
| PI(d18:0-22:5) | 0.55 | 0.67 | 1.05 | 0.55 |
| PI(d18:0-22:6) | 0.54 | 0.74 | 0.96 | 0.52 |
| PI(d18:1-18:1) | 0.41 | 0.29 | 1.13 | 0.68 |
| PI(d18:1-18:3) | 1.16 | 0.47 | 1.10 | 0.63 |
| PI(d18:1-20:4) | 0.81 | 0.96 | 0.99 | 0.53 |
| PI(d18:2-18:0) | 0.14 | 0.56 | 1.06 | 0.60 |
| PI(d18:2-18:1) | 0.24 | 0.33 | 1.16 | 0.64 |
| Arachidic Acid (20:0) Butyl Ester | 0.57 | 0.92 | 1.01 | 0.52 |
| Arachidonic Acid (20:4) Butyl Ester | 0.46 | 0.33 | 0.88 | 0.71 |
| Docosadienoic Acid (22:2) Butyl Ester | 1.36 | 0.69 | 1.02 | 0.52 |
| Docosahexaenoic Acid (22:6) Butyl Ester | 1.25 | 0.21 | 0.84 | 0.67 |
| Docosanoic Acid (22:0) Butyl Ester | 1.77 | 0.69 | 1.02 | 0.57 |
| Docosapentaenoic Acid (22:5) Butyl Ester | 1.12 | 0.35 | 0.95 | 0.64 |
| Docosatetraenoic Acid (22:4) Butyl Ester | 1.73 | 0.97 | 1.00 | 0.51 |
| Docosatrienoic Acid (22:3) Butyl Ester | 1.60 | 0.83 | 1.02 | 0.51 |
| Dotriacontanoic Acid (32:0) Butyl Ester | 7.20 | 0.22 | 1.27 | 0.71 |
| Eicosapentaenoic Acid (20:5) Butyl Ester | 0.71 | 0.45 | 0.95 | 0.61 |
| Eicosatrienoic Acid (20:1) Butyl Ester | 1.32 | 0.83 | 1.01 | 0.56 |
| Eicosatrienoic Acid (20:2) Butyl Ester | 1.39 | 0.41 | 0.95 | 0.64 |
| Eicosatrienoic Acid (20:3) Butyl Ester | 0.74 | 0.37 | 1.11 | 0.67 |
| Erucic Acid (22:1) Butyl Ester | 0.88 | 0.46 | 1.11 | 0.59 |
| Heptacosanoic Acid (27:0) Butyl Ester | 2.32 | 0.46 | 1.11 | 0.68 |
| Heptatriacontanoic Acid (37:0) Butyl Ester | 1.18 | 0.50 | 0.96 | 0.65 |
| Hexacosanoic Acid (26:0) Butyl Ester | 4.45 | 0.39 | 1.09 | 0.68 |
| Hexatriacontanoic Acid (36:0) Butyl Ester | 4.51 | 0.58 | 1.12 | 0.60 |
| Lauric acid (12:0) Butyl Ester | 1.25 | 0.33 | 0.84 | 0.73 |
| Lignoceric Acid (24:0) Butyl Ester | 2.33 | 0.87 | 0.99 | 0.53 |
| Linoleic Acid (18:2) Butyl Ester | 0.19 | 0.27 | 0.85 | 0.67 |
| Linolenic Acid (18:3) Butyl Ester | 0.35 | 0.52 | 0.93 | 0.61 |
| Myristic Acid (14:0) Butyl Ester | 0.81 | 0.37 | 0.95 | 0.61 |
| Nervonic Acid (24:1) Butyl Ester | 2.24 | 0.50 | 1.09 | 0.63 |
| Nonacosanoic Acid (29:0) Butyl Ester | 1.24 | 0.76 | 0.98 | 0.53 |
| Octacosanoic Acid (28:0) Butyl Ester | 20.01 | 0.35 | 1.34 | 0.63 |
| Oleic Acid (18:1) Butyl Ester | 0.63 | 0.24 | 0.87 | 0.69 |
| Palmitic Acid (16:0) Butyl Ester | 0.95 | 0.01 | 0.93 | 0.89 |
| Palmitoleic Acid (16:1) Butyl Ester | 0.99 | 0.24 | 0.84 | 0.63 |
| Pentacosanoic Acid (25:0) Butyl Ester | 1.64 | 0.11 | 1.18 | 0.73 |
| Stearic Acid (18:0) Butyl Ester | 1.04 | 0.01 | 0.96 | 0.84 |
| Tetratriacontanoic Acid (34:0) Butyl Ester | 2.91 | 0.25 | 1.17 | 0.64 |
| Triacontanoic Acid (30:0) Butyl Ester | 9.18 | 0.32 | 1.19 | 0.67 |
| Tritriacontanoic Acid (33:0) Butyl Ester | 1.15 | 0.41 | 0.95 | 0.61 |
| GalCer(d18:0-18:0) | 0.95 | 0.14 | 1.53 | 0.73 |
| GalCer(d18:0-20:0) | 0.98 | 0.88 | 1.03 | 0.51 |
| GalCer(d18:0-24:0) | 0.77 | 0.44 | 0.85 | 0.51 |
| GalCer(d18:1-16:0) | 1.34 | 0.83 | 1.03 | 0.55 |
| GalCer(d18:1-16:1) | 1.60 | 0.83 | 0.95 | 0.55 |
| GalCer(d18:1-18:0) | 0.97 | 0.34 | 0.82 | 0.67 |
| GalCer(d18:1-18:1) | 1.40 | 0.45 | 1.20 | 0.60 |
| GalCer(d18:1-20:0) | 0.51 | 0.92 | 1.03 | 0.52 |
| GalCer(d18:1-20:1) | 1.11 | 0.68 | 1.14 | 0.55 |
| GalCer(d18:1-22:0) | 0.33 | 0.34 | 1.22 | 0.63 |
| GalCer(d18:1-22:1) | 0.50 | 0.81 | 0.94 | 0.56 |
| GalCer(d18:1-22:2) | 0.37 | 0.77 | 1.08 | 0.53 |
| GalCer(d18:1-22:3) | 1.07 | 0.84 | 1.05 | 0.55 |
| GalCer(d18:1-23:1) | 0.88 | 0.73 | 1.08 | 0.57 |
| GalCer(d18:1-24:0) | 0.58 | 0.73 | 0.95 | 0.56 |
| GalCer(d18:1-24:1) | 0.60 | 0.32 | 1.18 | 0.71 |
| GalCer(d18:1-24:2) | 0.84 | 0.32 | 0.83 | 0.69 |
| LysoPC(a16:0) | 0.10 | 0.20 | 1.19 | 0.72 |
| LysoPC(a20:0) | 0.32 | 0.61 | 1.05 | 0.59 |
| LysoPC(a20:1) | 0.34 | 0.08 | 1.35 | 0.83 |
| LysoPC(a20:3) | 0.36 | 0.90 | 1.04 | 0.52 |
| LysoPC(a20:4) | 0.30 | 0.37 | 1.09 | 0.63 |
| LysoPC(a20:5) | 0.45 | 0.08 | 1.29 | 0.79 |
| LysoPC(a22:0) | 0.73 | 0.07 | 0.79 | 0.77 |
| LysoPC(a22:1) | 0.30 | 0.70 | 1.10 | 0.52 |
| LysoPC(a22:2) | 0.38 | 0.62 | 1.07 | 0.59 |
| LysoPC(a22:3) | 0.82 | 0.88 | 0.98 | 0.52 |
| LysoPC(a22:4) | 0.68 | 0.49 | 0.87 | 0.60 |
| LysoPC(a22:5) | 1.02 | 0.37 | 1.39 | 0.67 |
| LysoPC(a22:6) | 0.55 | 0.29 | 1.35 | 0.63 |
| LysoPC(a24:0) | 1.91 | 0.98 | 1.00 | 0.51 |
| LysoPC(d16:0) | 0.09 | 0.66 | 1.07 | 0.56 |
| LysoPC(d18:0) | 0.08 | 0.99 | 1.00 | 0.57 |
| LysoPC(d18:1) | 0.19 | 0.65 | 1.07 | 0.59 |
| LysoPC(d18:2) | 0.12 | 0.17 | 1.52 | 0.76 |
| LysoPC(d18:3) | 0.17 | 0.12 | 1.61 | 0.72 |
| LysoPC(d20:0) | 0.31 | 0.05 | 1.35 | 0.80 |
| LysoPC(d20:1) | 0.22 | 0.42 | 1.12 | 0.57 |
| LysoPC(d20:2) | 0.19 | 0.26 | 1.25 | 0.65 |
| LysoPC(d20:3) | 0.29 | 0.29 | 1.26 | 0.65 |
| LysoPC(d20:4) | 0.32 | 0.58 | 0.89 | 0.75 |
| LysoPC(d20:5) | 0.17 | 0.31 | 0.76 | 0.67 |
| LysoPC(d22:0) | 1.59 | 0.12 | 0.85 | 0.75 |
| LysoPC(d22:1) | 0.53 | 0.85 | 0.94 | 0.57 |
| LysoPC(d22:2) | 0.85 | 0.89 | 0.98 | 0.53 |
| LysoPC(d22:3) | 0.82 | 0.35 | 1.20 | 0.65 |
| LysoPC(d22:4) | 0.44 | 0.14 | 1.38 | 0.71 |
| LysoPC(d22:5) | 0.25 | 0.23 | 1.22 | 0.72 |
| LysoPC(d22:6) | 0.44 | 0.79 | 0.94 | 0.56 |
| LysoPC(d24:1) | 1.23 | 1.00 | 1.00 | 0.53 |
| LysoPC(d24:2) | 0.62 | 0.68 | 0.91 | 0.52 |
| LysoPC(p14:1) | 1.73 | 0.97 | 0.99 | 0.52 |
| LysoPC(p16:0) | 0.10 | 0.30 | 1.18 | 0.67 |
| LysoPC(p18:0) | 0.09 | 0.11 | 1.28 | 0.72 |
| LysoPC(p18:1)/LysoPC(a18:2) | 0.21 | 0.88 | 1.02 | 0.52 |
| LysoPC(p20:2) | 0.36 | 0.90 | 1.04 | 0.52 |
| LysoPC(p20:3) | 0.30 | 0.37 | 1.09 | 0.63 |
| LysoPC(p20:5) | 0.15 | 0.80 | 1.03 | 0.51 |
| LysoPC(p22:1) | 0.38 | 0.62 | 1.07 | 0.59 |
| PC(a14:0-20:2) | 0.70 | 0.96 | 1.01 | 0.56 |
| PC(a14:0-20:3) | 0.52 | 0.75 | 0.92 | 0.64 |
| PC(a14:0-22:5) | 0.56 | 0.26 | 0.76 | 0.68 |
| PC(a14:1-20:3)/PC(p14:0-20:3) | 0.65 | 0.64 | 1.09 | 0.63 |
| PC(a16:0-14:0) | 1.02 | 0.90 | 0.97 | 0.52 |
| PC(a16:0-18:0) | 1.99 | 0.24 | 1.27 | 0.69 |
| PC(a16:0-20:0) | 2.38 | 0.66 | 0.93 | 0.61 |
| PC(a16:0-22:0) | 0.32 | 0.30 | 0.79 | 0.63 |
| PC(a16:0-22:3) | 0.35 | 0.60 | 0.94 | 0.63 |
| PC(a16:0-22:4) | 1.53 | 0.07 | 1.51 | 0.80 |
| PC(a16:0-22:5) | 1.45 | 0.54 | 0.87 | 0.69 |
| PC(a16:0-22:6) | 1.00 | 0.92 | 1.02 | 0.55 |
| PC(a18:0-18:0) | 2.38 | 0.66 | 0.93 | 0.61 |
| PC(a18:0-18:1) | 0.83 | 0.20 | 1.19 | 0.65 |
| PC(a18:0-18:2) | 0.36 | 0.27 | 1.27 | 0.67 |
| PC(a18:0-18:3) | 0.36 | 0.67 | 1.06 | 0.53 |
| PC(a18:0-20:1) | 0.45 | 0.52 | 1.13 | 0.55 |
| PC(a18:0-20:4) | 0.65 | 0.74 | 0.94 | 0.71 |
| PC(a18:0-20:5) | 0.49 | 0.03 | 1.55 | 0.80 |
| PC(a18:0-22:0) | 0.35 | 0.92 | 0.98 | 0.51 |
| PC(a18:0-22:2)/PC(p18:0-22:1) | 0.59 | 0.36 | 1.13 | 0.57 |
| PC(a18:0-22:3)/PC(p18:0-22:2) | 0.35 | 0.72 | 1.06 | 0.55 |
| PC(a18:0-22:6) | 1.02 | 0.45 | 1.22 | 0.63 |
| PC(a18:3-20:2) | 0.41 | 0.52 | 1.12 | 0.60 |
| PC(a20:0-16:1) | 1.91 | 0.12 | 1.40 | 0.68 |
| PC(a20:0-18:3) | 1.19 | 0.94 | 1.01 | 0.51 |
| PC(a20:0-20:3) | 0.39 | 0.58 | 0.87 | 0.51 |
| PC(a20:0-20:5) | 1.35 | 0.61 | 1.11 | 0.55 |
| PC(a20:0-22:0) | 0.86 | 0.27 | 1.20 | 0.69 |
| PC(a20:0-22:1)/PC(p20:0-22:0) | 0.57 | 0.77 | 0.95 | 0.57 |
| PC(a20:1-16:1) | 0.71 | 0.87 | 1.04 | 0.53 |
| PC(a20:1-18:1) | 0.31 | 0.84 | 1.04 | 0.56 |
| PC(a20:2-16:0) | 1.70 | 0.33 | 1.25 | 0.67 |
| PC(a20:2-20:1) | 0.39 | 0.58 | 0.87 | 0.51 |
| PC(a22:0-20:2) | 0.39 | 0.37 | 0.77 | 0.63 |
| PC(a22:2-16:0) | 0.41 | 0.30 | 1.27 | 0.61 |
| PC(d12:0-14:1) | 0.04 | 0.85 | 0.96 | 0.60 |
| PC(d14:0-14:1) | 0.70 | 0.27 | 0.77 | 0.71 |
| PC(d14:0-16:0) | 1.17 | 0.17 | 1.16 | 0.72 |
| PC(d14:0-18:0) | 0.36 | 0.76 | 1.08 | 0.52 |
| PC(d14:0-18:1) | 0.59 | 0.05 | 1.46 | 0.83 |
| PC(d14:0-20:0) | 0.37 | 0.12 | 0.73 | 0.76 |
| PC(d14:0-20:3) | 0.34 | 0.18 | 1.37 | 0.76 |
| PC(d14:0-20:4) | 0.39 | 0.52 | 0.91 | 0.64 |
| PC(d14:1-18:0) | 0.48 | 0.87 | 0.96 | 0.55 |
| PC(d14:1-18:1) | 0.67 | 0.66 | 0.90 | 0.59 |
| PC(d14:1-18:3) | 1.65 | 0.97 | 1.01 | 0.60 |
| PC(d14:1-20:0) | 0.51 | 0.44 | 1.16 | 0.60 |
| PC(d14:1-20:2) | 0.73 | 0.85 | 0.97 | 0.53 |
| PC(d14:1-20:3) | 0.86 | 0.44 | 0.84 | 0.63 |
| PC(d14:1-20:5) | 0.77 | 0.82 | 0.95 | 0.52 |
| PC(d16:0-16:0) | 4.19 | 0.27 | 1.12 | 0.65 |
| PC(d16:0-16:3) | 0.87 | 0.98 | 1.00 | 0.52 |
| PC(d16:0-18:0) | 1.52 | 0.27 | 1.12 | 0.71 |
| PC(d16:0-18:1) | 1.23 | 0.27 | 1.14 | 0.76 |
| PC(d16:0-18:2) | 0.27 | 0.04 | 1.49 | 0.88 |
| PC(d16:0-18:3) | 0.33 | 0.27 | 1.38 | 0.65 |
| PC(d16:0-20:1) | 1.17 | 0.15 | 1.23 | 0.69 |
| PC(d16:0-20:2) | 0.52 | 0.03 | 1.41 | 0.89 |
| PC(d16:0-20:3) | 0.51 | 0.06 | 1.34 | 0.81 |
| PC(d16:0-20:4) | 0.56 | 0.45 | 0.91 | 0.68 |
| PC(d16:0-20:5) | 0.32 | 0.87 | 0.96 | 0.57 |
| PC(d16:0-22:2) | 1.34 | 0.98 | 1.01 | 0.51 |
| PC(d16:0-22:3) | 1.05 | 0.09 | 1.32 | 0.76 |
| PC(d16:0-22:4) | 0.47 | 0.20 | 1.19 | 0.67 |
| PC(d16:0-22:5) | 0.27 | 0.18 | 1.29 | 0.71 |
| PC(d16:0-22:6) | 0.52 | 0.59 | 0.92 | 0.52 |
| PC(d16:1-16:0) | 3.83 | 0.33 | 1.14 | 0.71 |
| PC(d16:1-16:1) | 0.87 | 0.65 | 1.09 | 0.57 |
| PC(d16:1-18:0) | 1.07 | 0.74 | 0.96 | 0.57 |
| PC(d16:1-18:1) | 0.86 | 0.79 | 1.04 | 0.53 |
| PC(d16:1-18:2) | 0.26 | 0.04 | 1.43 | 0.81 |
| PC(d16:1-18:3) | 0.47 | 0.07 | 0.66 | 0.67 |
| PC(d16:1-20:2) | 1.15 | 0.22 | 0.83 | 0.69 |
| PC(d16:1-20:3) | 0.41 | 0.80 | 0.96 | 0.52 |
| PC(d16:1-20:4) | 0.59 | 0.36 | 1.37 | 0.65 |
| PC(d16:1-20:5) | 0.47 | 0.76 | 0.91 | 0.59 |
| PC(d16:1-22:0) | 0.90 | 0.12 | 1.51 | 0.72 |
| PC(d16:1-22:2) | 0.76 | 0.83 | 0.95 | 0.57 |
| PC(d16:1-22:3) | 0.82 | 0.00 | 1.90 | 0.88 |
| PC(d16:1-22:5) | 1.33 | 0.33 | 0.83 | 0.61 |
| PC(d16:1-22:6) | 0.49 | 0.80 | 0.94 | 0.53 |
| PC(d16:2-18:0) | 0.69 | 0.70 | 1.08 | 0.51 |
| PC(d16:2-18:1) | 0.54 | 0.95 | 0.98 | 0.52 |
| PC(d16:2-20:0) | 1.04 | 0.42 | 1.13 | 0.51 |
| PC(d16:2-22:0) | 0.25 | 0.68 | 1.08 | 0.55 |
| PC(d16:2-22:2) | 0.89 | 0.71 | 0.93 | 0.64 |
| PC(d16:2-22:4) | 0.50 | 0.11 | 0.68 | 0.67 |
| PC(d16:2-22:6) | 0.92 | 0.95 | 0.99 | 0.52 |
| PC(d16:3-20:0) | 0.78 | 1.00 | 1.00 | 0.56 |
| PC(d16:3-20:1) | 0.56 | 0.75 | 0.95 | 0.64 |
| PC(d16:3-20:2) | 0.40 | 0.78 | 1.07 | 0.51 |
| PC(d16:3-20:4) | 0.72 | 0.17 | 1.29 | 0.73 |
| PC(d16:3-22:1) | 0.25 | 0.52 | 1.08 | 0.64 |
| PC(d16:3-22:2) | 0.42 | 0.65 | 1.09 | 0.52 |
| PC(d16:3-22:3) | 0.55 | 0.23 | 0.81 | 0.68 |
| PC(d16:3-22:4) | 0.53 | 0.26 | 1.32 | 0.65 |
| PC(d16:3-22:5) | 1.00 | 0.72 | 1.08 | 0.57 |
| PC(d18:0-18:0) | 0.72 | 0.22 | 1.17 | 0.57 |
| PC(d18:0-18:1) | 0.78 | 0.26 | 1.11 | 0.72 |
| PC(d18:0-18:2) | 0.16 | 0.11 | 1.34 | 0.77 |
| PC(d18:0-18:3) | 0.36 | 0.28 | 0.82 | 0.69 |
| PC(d18:0-20:0) | 1.17 | 0.26 | 1.25 | 0.65 |
| PC(d18:0-20:1) | 0.89 | 0.64 | 0.92 | 0.60 |
| PC(d18:0-20:2) | 0.30 | 0.75 | 1.06 | 0.57 |
| PC(d18:0-20:3) | 0.32 | 0.15 | 1.19 | 0.71 |
| PC(d18:0-20:4) | 0.43 | 0.25 | 0.87 | 0.72 |
| PC(d18:0-20:5) | 0.23 | 0.88 | 1.04 | 0.51 |
| PC(d18:0-22:0) | 0.51 | 0.80 | 0.96 | 0.64 |
| PC(d18:0-22:1) | 0.26 | 0.70 | 0.96 | 0.55 |
| PC(d18:0-22:2) | 0.54 | 0.19 | 1.35 | 0.73 |
| PC(d18:0-22:3) | 0.57 | 0.42 | 0.85 | 0.63 |
| PC(d18:0-22:4) | 0.27 | 0.11 | 1.34 | 0.77 |
| PC(d18:0-22:5) | 0.22 | 0.70 | 1.07 | 0.53 |
| PC(d18:0-22:6) | 0.36 | 0.77 | 0.95 | 0.56 |
| PC(d18:0-24:0) | 0.95 | 0.48 | 1.16 | 0.57 |
| PC(d18:0-24:1) | 0.53 | 0.89 | 1.02 | 0.51 |
| PC(d18:1-18:1) | 0.66 | 0.38 | 1.16 | 0.63 |
| PC(d18:1-18:2) | 0.13 | 0.10 | 1.60 | 0.83 |
| PC(d18:1-20:0) | 0.63 | 0.37 | 1.26 | 0.63 |
| PC(d18:1-20:1) | 0.94 | 0.97 | 1.01 | 0.51 |
| PC(d18:1-20:2) | 0.32 | 0.95 | 1.02 | 0.52 |
| PC(d18:1-20:3) | 0.32 | 0.06 | 1.38 | 0.77 |
| PC(d18:1-20:4) | 0.37 | 0.98 | 1.00 | 0.53 |
| PC(d18:1-20:5) | 0.31 | 0.63 | 0.91 | 0.56 |
| PC(d18:1-22:0) | 1.24 | 0.48 | 1.21 | 0.65 |
| PC(d18:1-22:1) | 1.04 | 0.04 | 1.33 | 0.85 |
| PC(d18:1-22:2) | 0.99 | 0.77 | 0.94 | 0.59 |
| PC(d18:1-22:3) | 0.97 | 0.29 | 1.28 | 0.75 |
| PC(d18:1-22:4) | 0.79 | 0.17 | 1.32 | 0.65 |
| PC(d18:1-22:6) | 0.44 | 0.49 | 0.89 | 0.56 |
| PC(d18:2-18:2) | 0.76 | 0.73 | 0.86 | 0.51 |
| PC(d18:2-18:3) | 0.62 | 0.78 | 1.05 | 0.51 |
| PC(d18:2-20:0) | 0.59 | 0.99 | 1.00 | 0.52 |
| PC(d18:2-20:1) | 0.45 | 0.19 | 1.35 | 0.64 |
| PC(d18:2-20:2) | 0.41 | 0.78 | 1.05 | 0.65 |
| PC(d18:2-20:4) | 0.20 | 0.64 | 0.93 | 0.56 |
| PC(d18:2-22:0) | 0.53 | 0.42 | 1.14 | 0.65 |
| PC(d18:2-22:3) | 1.25 | 0.44 | 1.15 | 0.64 |
| PC(d18:2-22:6) | 0.47 | 0.52 | 1.17 | 0.60 |
| PC(d18:3-20:0) | 0.58 | 0.41 | 1.16 | 0.57 |
| PC(d18:3-20:1) | 0.41 | 0.52 | 0.88 | 0.59 |
| PC(d18:3-20:4) | 1.48 | 0.70 | 1.10 | 0.56 |
| PC(d18:3-20:5) | 1.73 | 0.59 | 1.12 | 0.53 |
| PC(d18:3-22:2) | 0.74 | 0.77 | 1.06 | 0.52 |
| PC(d18:4-22:2) | 0.59 | 0.97 | 1.01 | 0.53 |
| PC(d20:0-20:5) | 0.84 | 0.38 | 1.17 | 0.63 |
| PC(d20:1-20:2) | 0.82 | 0.84 | 1.04 | 0.52 |
| PC(d20:1-20:4) | 0.61 | 0.05 | 0.76 | 0.84 |
| PC(d20:1-20:5) | 0.43 | 0.25 | 1.33 | 0.71 |
| PC(d20:1-22:2) | 1.15 | 0.80 | 1.04 | 0.51 |
| PC(d20:1-22:5) | 0.42 | 0.34 | 1.29 | 0.64 |
| PC(d20:1-22:6) | 0.64 | 0.09 | 1.32 | 0.75 |
| PC(d20:2-20:2) | 0.93 | 0.89 | 0.97 | 0.56 |
| PC(d20:2-20:4) | 0.53 | 0.41 | 0.88 | 0.63 |
| PC(d20:2-20:5) | 0.50 | 0.71 | 1.10 | 0.59 |
| PC(d20:2-22:0) | 0.63 | 0.43 | 0.91 | 0.63 |
| PC(d20:2-22:3) | 0.45 | 0.51 | 0.88 | 0.71 |
| PC(d20:2-22:6) | 0.78 | 0.22 | 1.19 | 0.64 |
| PC(d20:2-24:1) | 0.80 | 0.06 | 0.62 | 0.73 |
| PC(d20:3-20:5) | 0.41 | 0.14 | 1.56 | 0.71 |
| PC(d20:4-20:4) | 0.79 | 0.64 | 0.90 | 0.63 |
| PC(d20:5-22:2) | 0.57 | 0.05 | 1.72 | 0.84 |
| PC(d20:5-22:3) | 0.57 | 0.77 | 0.95 | 0.57 |
| PC(d20:5-22:4) | 0.53 | 0.44 | 1.16 | 0.64 |
| PC(d20:5-22:5) | 0.38 | 0.43 | 0.84 | 0.68 |
| PC(d20:5-24:1) | 0.51 | 0.47 | 0.86 | 0.57 |
| PC(d22:3-22:6) | 0.74 | 0.85 | 0.95 | 0.52 |
| PC(d22:6-22:6) | 0.61 | 0.46 | 1.19 | 0.56 |
| PC(p14:0-18:0)/PC(a14:1-18:0) | 1.02 | 0.84 | 0.96 | 0.52 |
| PC(p14:0-20:5) | 0.92 | 0.27 | 0.85 | 0.56 |
| PC(p14:1-18:2) | 0.72 | 0.13 | 0.76 | 0.68 |
| PC(p14:1-20:5) | 0.20 | 0.44 | 0.89 | 0.56 |
| PC(p16:0-16:0) | 1.36 | 0.15 | 1.35 | 0.68 |
| PC(p16:1-18:2) | 0.43 | 0.95 | 0.99 | 0.53 |
| PC(p16:1-22:0) | 1.43 | 0.13 | 1.21 | 0.69 |
| PC(p16:2-16:0) | 0.83 | 0.70 | 1.05 | 0.60 |
| PC(p16:2-18:0) | 0.57 | 0.48 | 1.09 | 0.67 |
| PC(p16:2-18:1) | 1.11 | 0.25 | 1.14 | 0.72 |
| PC(p16:2-18:2) | 0.77 | 0.63 | 1.07 | 0.63 |
| PC(p16:2-20:0) | 3.40 | 0.50 | 1.10 | 0.69 |
| PC(p16:2-20:1) | 0.66 | 0.22 | 1.16 | 0.73 |
| PC(p16:2-20:2) | 0.63 | 0.66 | 1.05 | 0.52 |
| PC(p16:2-20:3) | 0.28 | 0.73 | 0.96 | 0.57 |
| PC(p16:2-20:4) | 0.80 | 0.75 | 1.04 | 0.55 |
| PC(p16:2-22:0) | 1.34 | 0.14 | 1.18 | 0.83 |
| PC(p16:2-22:2) | 0.66 | 0.20 | 1.21 | 0.73 |
| PC(p16:2-22:3) | 0.40 | 0.54 | 1.07 | 0.61 |
| PC(p16:2-22:4) | 0.40 | 0.76 | 1.04 | 0.56 |
| PC(p16:2-22:5) | 0.28 | 0.64 | 1.06 | 0.56 |
| PC(p16:2-22:6) | 0.54 | 0.34 | 1.08 | 0.71 |
| PC(p16:2-24:0) | 0.69 | 0.01 | 1.32 | 0.87 |
| PC(p16:2-24:1) | 0.60 | 0.01 | 1.32 | 0.89 |
| PC(p16:2-24:2) | 0.56 | 0.04 | 1.33 | 0.87 |
| PC(p16:3-22:4) | 0.57 | 0.01 | 0.63 | 0.88 |
| PC(p16:3-22:5) | 0.43 | 0.63 | 0.88 | 0.52 |
| PC(p18:0-16:0) | 1.13 | 0.99 | 1.00 | 0.51 |
| PC(p18:0-22:5) | 1.02 | 0.45 | 1.22 | 0.63 |
| PC(p18:1-20:2) | 0.90 | 0.45 | 1.18 | 0.68 |
| PC(p18:2-16:0) | 0.65 | 0.45 | 0.87 | 0.63 |
| PC(p18:2-18:1) | 0.79 | 0.26 | 1.17 | 0.65 |
| PC(p18:2-18:2) | 1.08 | 0.05 | 1.39 | 0.80 |
| PC(p18:2-18:3) | 3.10 | 0.56 | 1.11 | 0.55 |
| PC(p18:2-20:1) | 0.33 | 0.02 | 1.56 | 0.93 |
| PC(p18:2-20:3) | 0.23 | 0.63 | 1.05 | 0.56 |
| PC(p18:2-22:0) | 0.88 | 0.28 | 1.10 | 0.59 |
| PC(p18:2-22:1) | 0.19 | 0.13 | 1.27 | 0.77 |
| PC(p18:2-22:2) | 0.33 | 0.05 | 1.33 | 0.84 |
| PC(p18:2-22:3) | 0.29 | 0.97 | 1.00 | 0.51 |
| PC(p18:2-22:4) | 0.27 | 0.07 | 0.82 | 0.76 |
| PC(p18:2-22:5) | 0.21 | 0.54 | 1.09 | 0.61 |
| PC(p18:2-24:0) | 0.39 | 0.14 | 1.28 | 0.63 |
| PC(p18:2-24:1) | 0.44 | 0.04 | 1.22 | 0.83 |
| PC(p18:2-24:2) | 0.37 | 0.14 | 1.20 | 0.72 |
| PC(p18:3-16:2) | 0.84 | 0.51 | 1.09 | 0.57 |
| PC(p18:3-18:0) | 0.78 | 0.08 | 1.46 | 0.76 |
| PC(p18:3-20:0) | 1.35 | 0.68 | 1.08 | 0.60 |
| PC(p18:3-22:0) | 0.75 | 0.03 | 1.44 | 0.88 |
| PC(p18:3-22:1) | 0.16 | 0.12 | 1.52 | 0.77 |
| PC(p18:3-22:4) | 0.42 | 0.76 | 1.05 | 0.52 |
| PC(p18:3-24:1) | 0.66 | 0.07 | 1.31 | 0.72 |
| PC(p18:3-24:2) | 0.37 | 0.07 | 1.53 | 0.77 |
| PC(p18:4-20:1) | 0.40 | 0.63 | 1.12 | 0.59 |
| PC(p18:4-22:0) | 0.41 | 0.06 | 1.32 | 0.80 |
| PC(p18:4-22:1) | 0.11 | 0.07 | 1.65 | 0.83 |
| PC(p18:4-22:2) | 0.33 | 0.75 | 1.07 | 0.53 |
| PC(p18:4-22:3) | 0.21 | 0.20 | 1.23 | 0.68 |
| PC(p18:4-22:4) | 0.22 | 0.43 | 1.26 | 0.63 |
| PC(p18:4-22:5) | 0.18 | 0.94 | 1.02 | 0.57 |
| PC(p18:4-22:6) | 0.21 | 0.22 | 1.28 | 0.61 |
| PC(p20:0-16:0) | 0.78 | 0.21 | 1.30 | 0.69 |
| PC(p20:2-22:6) | 0.49 | 0.06 | 0.69 | 0.81 |
| PC(p20:3-22:6) | 0.77 | 0.11 | 1.46 | 0.68 |
| PC(p20:4-18:1) | 1.41 | 0.67 | 1.07 | 0.52 |
| PC(p20:4-22:5) | 0.56 | 0.97 | 1.00 | 0.52 |
| PC(p20:4-22:6) | 0.65 | 0.68 | 1.05 | 0.53 |
| PC(p20:5-22:0) | 0.67 | 0.26 | 1.30 | 0.65 |
| PC(p22:0-22:1) | 0.37 | 0.43 | 1.15 | 0.59 |
| PC(p22:0-24:0) | 0.85 | 0.24 | 1.39 | 0.65 |
| PC(p22:1-22:6) | 0.88 | 0.25 | 1.15 | 0.65 |
| PC(p22:2-20:2) | 1.30 | 0.94 | 0.99 | 0.55 |
| PC(p22:4-20:2) | 0.70 | 0.34 | 1.29 | 0.71 |
| PC(p22:6-18:2) | 0.59 | 0.99 | 1.00 | 0.52 |
| SM(d18:0-22:0) | 0.87 | 0.00 | 0.79 | 0.92 |
| SM(d18:0-24:0) | 3.22 | 0.03 | 0.84 | 0.81 |
| SM(d18:1-14:0) | 1.06 | 0.78 | 0.97 | 0.56 |
| SM(d18:1-14:1) | 0.83 | 0.13 | 0.88 | 0.72 |
| SM(d18:1-15:0) | 1.14 | 0.89 | 1.01 | 0.53 |
| SM(d18:1-15:1) | 0.83 | 0.03 | 0.74 | 0.76 |
| SM(d18:1-16:0) | 1.36 | 0.69 | 0.97 | 0.59 |
| SM(d18:1-16:1) | 0.94 | 0.45 | 0.94 | 0.61 |
| SM(d18:1-17:0) | 1.50 | 0.39 | 0.91 | 0.61 |
| SM(d18:1-17:1) | 1.53 | 0.17 | 0.85 | 0.68 |
| SM(d18:1-18:0) | 1.53 | 0.25 | 0.90 | 0.65 |
| SM(d18:1-18:1) | 1.82 | 0.24 | 0.87 | 0.67 |
| SM(d18:1-18:2) | 0.48 | 0.70 | 0.95 | 0.51 |
| SM(d18:1-18:3) | 1.44 | 0.32 | 2.05 | 0.67 |
| SM(d18:1-19:0) | 1.03 | 0.39 | 0.91 | 0.56 |
| SM(d18:1-19:1) | 1.85 | 0.40 | 0.89 | 0.61 |
| SM(d18:1-20:0) | 0.53 | 0.13 | 0.88 | 0.72 |
| SM(d18:1-20:1) | 1.17 | 0.27 | 0.89 | 0.64 |
| SM(d18:1-20:2) | 0.68 | 0.63 | 0.94 | 0.57 |
| SM(d18:1-20:3) | 0.79 | 0.60 | 0.89 | 0.59 |
| SM(d18:1-20:5) | 2.07 | 0.60 | 0.85 | 0.61 |
| SM(d18:1-21:0) | 0.26 | 0.89 | 1.02 | 0.55 |
| SM(d18:1-21:1) | 0.59 | 0.06 | 0.78 | 0.84 |
| SM(d18:1-22:0) | 0.85 | 0.07 | 0.87 | 0.77 |
| SM(d18:1-22:1) | 0.48 | 0.29 | 0.91 | 0.68 |
| SM(d18:1-22:2) | 0.70 | 0.40 | 0.91 | 0.63 |
| SM(d18:1-22:3) | 0.95 | 0.37 | 1.14 | 0.63 |
| SM(d18:1-22:4) | 2.29 | 0.74 | 0.94 | 0.56 |
| SM(d18:1-22:6) | 0.57 | 0.28 | 0.83 | 0.69 |
| SM(d18:1-23:0) | 0.29 | 0.20 | 0.88 | 0.68 |
| SM(d18:1-23:1) | 0.28 | 0.66 | 0.96 | 0.59 |
| SM(d18:1-23:2) | 0.65 | 0.74 | 0.95 | 0.52 |
| SM(d18:1-24:0) | 3.01 | 0.14 | 0.89 | 0.75 |
| SM(d18:1-24:1) | 1.48 | 0.62 | 1.05 | 0.61 |
| SM(d18:1-24:2) | 0.86 | 0.75 | 0.97 | 0.57 |
| SM(d18:1-24:3) | 0.91 | 0.29 | 0.89 | 0.63 |
| SM(d18:1-25:0) | 2.05 | 0.76 | 0.96 | 0.56 |
| SM(d18:1-25:1) | 1.71 | 0.31 | 1.12 | 0.67 |
| SM(d18:1-25:2) | 2.22 | 0.99 | 1.00 | 0.53 |
| SM(d18:1-26:0) | 15.63 | 0.08 | 0.88 | 0.76 |
| SM(d18:1-26:1) | 12.40 | 0.57 | 0.94 | 0.64 |
| SM(d18:1-26:2) | 7.45 | 0.91 | 1.01 | 0.53 |

CE: Cholesterol esters. TG: Triglyceride. DG: Diglyceride. Cer: Ceramide. CerP: Ceramide-1-phosphate. LysoPA: Lysophosphatidic acid. LysoPE: lysophosphatidylethanolamine. PI: Phosphatidylinositol. GalCer: Galactosylceramide. PC: Phosphatidylcholine. SM: Sphingomyelin.

References

1. Zhu, L., et al., *Serological Phenotyping Analysis Uncovers a Unique Metabolomic Pattern Associated With Early Onset of Type 2 Diabetes Mellitus.* Front Mol Biosci, 2022. **9**: p. 841209.

2. Kanehisa, M., et al., *KEGG: integrating viruses and cellular organisms.* Nucleic Acids Res, 2021. **49**(D1): p. D545-D551.

3. Fahy, E., et al., *Update of the LIPID MAPS comprehensive classification system for lipids.* J Lipid Res, 2009. **50 Suppl**(Suppl): p. S9-14.

4. Bradshaw, C.J.A. and S. Herrando-Perez, *Logistic-growth models measuring density feedback are sensitive to population declines, but not fluctuating carrying capacity.* Ecol Evol, 2023. **13**(4): p. e10010.

5. Peng, Y., et al., *LGBMDF: A cascade forest framework with LightGBM for predicting drug-target interactions.* Front Microbiol, 2022. **13**: p. 1092467.

6. Ke, G., et al., *LightGBM: a highly efficient gradient boosting decision tree*, in *Proceedings of the 31st International Conference on Neural Information Processing Systems*. 2017, Curran Associates Inc.: Long Beach, California, USA. p. 3149–3157.
